# Factors affecting the mercury concentration in the hair of young residents of the Vologda region, Russia

**DOI:** 10.1016/j.heliyon.2020.e04580

**Published:** 2020-08-05

**Authors:** Iuliia Aleksina, Viktor Komov

**Affiliations:** a Cherepovets State University, Cherepovets, Vologda Region, 162600, Russia; b Papanin Institute for Biology of Inland Waters, Russian Academy of Sciences, Borok, Yaroslavl Region, 152742, Russia

**Keywords:** Total mercury, Human scalp hair, Students, Region of residence, Fish consumption, Ecological health, Global ecological change, Environmental impact assessment, Environmental pollution, Environmental risk assessment, Environmental toxicology, Environmental science, Public health, Toxicology

## Abstract

The main aim of this study was to assess the level of mercury accumulation in the hair of students studying in the Vologda Region, Russia. Particular attention is devoted to clarifying the relationship between the metal concentration in the hair and the amount of fish in the diet. It was found that the mercury content in the hair of Vologda Region residents aged 17–21 is in the range from less than 0.002 mg/kg to 1.69 mg/kg, the median of the indicator for the entire sample is 0.14 mg/kg. The median value of mercury content in the hair of males (0.12 mg/kg) is lower than in the hair of females (0.16 mg/kg). The lowest metal concentrations were observed in individuals living in the city of Cherepovets and the highest metal concentration among the entire sample and in females in the western areas, and males in the eastern areas of the Vologda Region. The concentration of metal in the hair of the urban population is lower than in subjects living in rural areas. A higher metal content in the hair was recorded in individuals whose diet usually contains fish twice a month.

## 1 Introduction

The negative impact of mercury (Hg) on public health in recent decades remains a significant problem in many countries (Clarkson et al., 2003; UNEP, 2013; Sheehan et al., 2014). The greatest danger is the organic form - methylmercury (MeHg), which easily accumulates in aquatic organisms and is transmitted along the food chain to higher trophic levels. It is obvious that mercury presence in fish and other wild animals has risk for the organisms themselves as well as for their consumers (Kidd et al., 2012).

The presence of fish with a high mercury content in the diet disrupts the reproduction of some bird species and negatively affects the higher nervous activity of mammals (Scheuhammer et al., 2007). At the same time, the consumption of fish and seafood is the main source of heavy metal intake into the human body (Trasande et al., 2005; Scheuhammer et al., 2007; Karagas et al., 2012; Choi and Grandjean, 2012; Soon et al., 2014). When ingested with food, about 95% of methylmercury is absorbed through the gastrointestinal tract and distributed throughout the body (WHO, 2007). The results of studies evaluating the effects of mercury and its forms on human health indicate a neurotoxic effect and a high risk of developing cardiovascular and neurological diseases (Valera et al., 2008; Mozaffarian et al., 2011; Azevedo et al., 2012).

High mercury concentrations in fish can be recorded in regions that do not have local natural and anthropogenic sources of mercury emissions due to its global transportation with air masses (Poulin and Gibb, 2008). So over the past decades in the Vologda Region, a high metal content in soil, earthworms, fish, organs of small and medium mammals, and pet hair has been regularly recorded (Haines et al., 1995; Komov et al., 2017, 2016; 2004; Bachina et al., 2018). In some lakes and reservoirs from western regions, the mercury content in fish muscles was noted (Nemova et al., 2014), exceeding the sanitary and epidemiological standard levels of 0.3 and 0.6 mg/kg for freshwater non-predatory and predatory species, respectively (SanPiN 2.3.2.1078-01, 2011).

According to the Federal State Statistics Service, among 10 regions of the North-West Federal District, the Vologda Region takes third place in terms of the number of fish consumed after the Arkhangelsk Region (including the Nenets Autonomous district) and the Republic of Karelia. In 2016, the consumption of fish and fish products per household member in the region amounted to 22.4 kg/year, which exceeded the indicators obtained in 2015 (20.9 kg/year) ([dataset] Household food consumption in 2017, 2018). Despite the increase in fish consumption, information on the level of heavy metal among the population of the region, including its level in the hair, is fragmentary (Shuvalova et al., 2018; Rumiantseva et al., 2018).

Currently, the main attention is paid to studying the effect of mercury on the health status of the population most vulnerable to environmental factors; among them: children and women of childbearing age (Pesch et al., 2002; McDowell et al., 2004; Pinheiro et al., 2005; Raymond et al., 2017); individuals engaged in artisanal and small-scale gold mining, as well as living close to gold mining sites (Sheehan et al., 2014; Langeland et al., 2017); individuals consuming fish and seafood (Hajeb et al., 2008; Thapa et al., 2014; Soon et al., 2014).

In this context, it seemed appropriate to assess the level of metal accumulation in the hair of students studying in the Vologda Region (Russia), since the health status of this category of people may be a harbinger of changes in public health in subsequent years (Rosenfeld and Batrymbetova, 2008) and is recognized as a “resource” providing social status, effective employment and material well-being (Leonidova et al., 2015). The main evaluation criterion in our study was the relationship between the content of mercury in the hair and the amount of fish in the diet.

## 2 Material and methods

The study involved 412 students of the Cherepovets State University (hereinafter - ChSU, University), enrolled in the 1st year in 2016 at the age of 17–21 years (149 males and 263 females); of these: 258 students permanently resided in the territory of the city of Cherepovets, and 154 were visitors from other areas of the Vologda Region, and at the time of the study they were in the city for less than one month. The research protocol was reviewed and approved, prior to its conduct, by the Ethics Committee of the Cherepovets State University.

In determining the role of fish as a source of metal intake in the human body, measurement of the total mercury content in hair is widely used (WHO, 2015; UNEP, 2013). Hair samples were collected at the beginning of the study year, from September 1 to September 30, 2016 with the consent of the subjects in accordance with the Ethical Principles of Medical Research with the participation of people as subjects of the study of the Helsinki Declaration (World Medical Association, 2008). All subjects were acquainted with the purpose, methods and conditions of voluntary participation in the study, as well as the principles of confidentiality.

A University medical worker selected a strand of hair from the occipital part of the head for analysis (using previously sterilized stainless steel scissors), a few millimeters thick (at least 0.1 g), from the roots (Aleksina, 2017). Information on the region of residence, age, gender, and dietary habits of students was obtained using questionnaires. The collected material (hair samples) was stored in a paper envelope attached to the participant's application form.

In the hair samples, the mercury content was determined using a RA-915M mercury analyzer and a PIRO-915 + (Lumex-Marketing, St. Petersburg, Russia) pyrolytic console without preliminary preparation on the basis of the Ecological and Analytical Laboratory of the Department of Biology of ChSU. The technical capabilities of the analyzer allow reaching the detection limit of 0.002 mg/kg. Before analysis, each sample was divided into several strands and weighed in a quartz spoon on an analytical scale without dividing into segments (from roots to ends), then a weighed portion was moistened with bidistilled water in order to exclude the possibility of sample loss. Next, measurements were made in duplicate. As a result, the arithmetic average of two measurements was fixed. Operational control of the measurement error of the device was carried out by using certified biological material DORM-2 (certified value 4.47 ± 0.032 mg/kg, Institute of Environmental Chemistry, Ottawa, Canada) after every 10 samples. The average concentration of mercury determined in the certified sample was 4.436 mg/kg, coefficient of variation - 7.77%.

Analysis of the results was carried out both considering all the samples and the samples of males and females separately. Between the studied samples, the following groups were distinguished: by district of residence - permanently living in the city of Cherepovets - the largest industrial center of the North-West of Russia (Industry of the city of Cherepovets, 2018) and living in the western and eastern areas of the Vologda Region; according to the frequency of fish consumption - consuming fish several times a year and more often than twice a month; as well as at the place of residence - rural (all subjects, with the exception of residents of the city of Cherepovets and Vologda) and urban population (residents of the city of Cherepovets and Vologda).

Statistical analysis of the results was carried out using the software Statistica 12.0 (StatSoft Inc., USA). To assess the normality of the distribution of data on the mercury content in the hair, the Kolmogorov-Smirnov and Shapiro-Wilk criteria were used, and the asymmetry and excess coefficients were calculated. Since the metal concentration in the test material was uneven, a logarithmic transformation was used. The logarithm procedure also did not lead to a normal distribution of indicators, which determined the use of nonparametric statistical methods.

The results of the study are presented (Table 1) as the median (Me) and the corresponding 25 and 75 quartiles (Q25, Q75). The data obtained were compared with available publications; for this, the following indicators were calculated: arithmetic mean value (Mean), standard deviation (SD), range (R), quartile range (QR), minimum (Min) and maximum (Max) values. Comparison in paired groups (gender, place of residence and fish consumption) was carried out using the nonparametric Mann-Whitney U-test, the differences between the three groups (region of residence) were evaluated using the Kruskal-Wallis test. P < 0.05 was taken as the level of statistical significance.

**Table 1:** The mercury content in the hair of residents of the Vologda Region (N = 412) aged 17–21 years. Mercury concentrations are reported as mg/kg.

Sample characteristics	N, %	Mercury concentration in hair, mg/kg								
		Mean	Me	Min	Max	Q25	Q75	QR	SD	SE
Whole sample	100	0.20	0.14	<0.002	1.69	0.08	0.24	0.16	0.21	0.01
Gender, *p = 0.005∗*										

Males	36	0.19	0.12^a^	<0.002	1.69	0.06	0.22	0.16	0.25	0.02
Females	64	0.21	0.16^b^	<0.002	1.35	0.09	0.25	0.16	0.19	0.01

Place of residence, *p = 0.016∗*										

Village	34	0.22	0.15^b^	<0.002	1.35	0.1	0.28	0.18	0.21	0.02
City	66	0.19	0.13^a^	<0.002	1.69	0.07	0.22	0.15	0.22	0.01

Region of residence, ^*KW*^*p = 0.023∗∗*										

Cherepovets	63	0.19	0.13^a^	<0.002	1.69	0.07	0.22	0.16	0.22	0.01
Western areas	18	0.26	0.18^b^	<0.002	1.35	0.1	0.35	0.26	0.26	0.03
Eastern areas	19	0.19	0.15^ab^	<0.002	0.65	0.1	0.23	0.13	0.13	0.02

Fish consumption, *p = 0.000∗*										

More than 2 times per months	62	0.25	0.17^a^	0.01	1.69	0.11	0.31	0.2	0.24	0.02
A Few times per year	38	0.13	0.09^b^	<0.002	0.83	0.05	0.17	0.12	0.14	0.01

∗ - p values obtained using the Mann-Whitney U-test are significant at p < 0.05; ∗∗ ^*KW*^*p* — - p values obtained using the Kruskal-Wallis test are significant at p < 0.05.

a the value of the indicator is significantly lower than *b*.

b the value of the indicator is significantly higher than *a*.

ab the value of the indicator does not differ from *a* or *b*.

## 3 Results

The mercury content in the hair of Vologda Region residents aged 17–21 is in the range from less 0.002 mg/kg to 1.69 mg/kg (Table 1), the median of the indicator for the entire sample (N = 412) is 0.14 (0.08–0.24) mg/kg (for further details, please see the Supplementary Material.pdf file).

The concentration of metal in the hair of the study participants living in urban areas is significantly lower (Mann-Whitney U, U = 2.41, p = 0.016) than those living in rural areas. Furthermore indicators of the level of metal in males are lower (Mann-Whitney U, U = 2.79, p = 0.005) than indicators recorded in females.

When comparing the values of mercury content in the hair of subjects from different regions of the Vologda Region, statistically significant differences (Kruskal-Wallis, H = 7.53, df = 2, p = 0.023) were found between representatives of the western areas and the city of Cherepovets. The results recorded in representatives of the eastern areas of the region do not significantly differ from the results of peers living in the study area from the western areas and the city of Cherepovets and the city of Vologda.

The values of the mercury content in the hair of young men (Table 2) living in different territories of the region do not statistically differ in the city or village (Kruskal-Wallis, H = 1.54, df = 2, p = 0.463 and Mann-Whitney U, U = 1.17, p = 0.241, respectively).

**Table 2:** Mercury content in the hair of males living in the Vologda Region (N = 149) aged 17–21. Mercury concentrations are reported as mg/kg.

Sample characteristics	N, %	Mercury concentration in hair, mg/kg								
		Mean	Me	Min	Max	Q25	Q75	QR	SD	SE
Place of residence, *p = 0.241*										

Village	32	0.19	0.142	<0.002	1.03	0.06	0.25	0.19	0.18	0.03
City	68	0.2	0.1	<0.002	1.69	0.05	0.2	0.15	0.28	0.03

Region of residence, ^*KW*^*p = 0.463*										

Cherepovets	66	0.2	0.1	<0.002	1.69	0.05	0.2	0.15	0.28	0.03
Western areas	20	0.2	0.13	0.01	1.03	0.08	0.25	0.17	0.218	0.04
Eastern areas	14	0.18	0.15	<0.002	0.54	0.06	0.23	0.17	0.16	0.04

Fish consumption, *p = 0.000∗*										

More than 2 times per months	64	0.25	0.15^b^	0.01	1.69	0.09	0.32	0.23	0.29	0.03
Few times per year	36	0.10	0.06^a^	<0.002	0.71	0.03	0.12	0.09	0.12	0.02

∗ - p values obtained using the Mann-Whitney U-test are significant at p < 0.05; ^*KW*^*p* — p values obtained using the Kruskal-Wallis test are significant at p < 0.05.

a the value of the indicator is significantly lower than *b*.

b the value of the indicator is significantly higher than *a*.

For females living in an urban environment, the metal concentration in the test material is lower (Mann-Whitney U, U = -1.99, p = 0.046) than for participants living in rural areas, but the mercury content in the hair of the representatives of the western parts of the region is higher (Kruskal-Wallis, H = 6.73, df = 2, p = 0.035) than among the young women of the representatives of the urban population (Table 3).

**Table 3:** Mercury content in the hair of females living in the Vologda Region (N = 263) aged 17–21 years. Mercury concentrations are reported as mg/kg.

Sample characteristics	N, %	Mercury concentration in hair, mg/kg								
		Mean	Me	Min	Max	Q25	Q75	QR	SD	SE
Place of residence, *p = 0.046∗*										

Village	36	0.24	0.17^b^	<0.002	1.35	0.11	0.3	0.2	0.22	0.02
City	64	0.19	0.15^a^	<0.002	0.84	0.08	0.22	0.14	0.17	0.01

Region of residence, ^*KW*^*р = 0.035∗∗*										

Cherepovets	60	0.19	0.14^a^	<0.002	0.84	0.08	0.23	0.15	0.17	0.01
Western areas	18	0.29	0.20^b^	<0.002	1.35	0.11	0.39	0.28	0.28	0.04
Eastern areas	22	0.19	0.15^ab^	0.04	0.65	0.11	0.22	0.12	0.12	0.02

Fish consumption, *p = 0.000∗*										

More than 2 times per months	61	0.25	0.19^b^	0.01	1.35	0.11	0.31	0.19	0.2	0.02
Few times per year	39	0.15	0.11^a^	<0.002	0.83	0.06	0.18	0.12	0.15	0.02

∗ - p values obtained using the Mann-Whitney U-test are significant at p < 0.05; ∗∗ ^*KW*^*p* - p values obtained using the Kruskal-Wallis test are significant at p < 0.05.

a the value of the indicator is significantly lower than *b*.

b the value of the indicator is significantly higher than *a*.

ab the value of the indicator does not differ from *a* or *b*.

An analysis of the relationship between the mercury content in the hair and the frequency of fish consumption showed that males and females, who in their diet have fish more than twice a month (62%), have a higher mercury content in their hair (Mann-Whitney U, U = 7.08, p = 0,000) than those who practically excluded fish from their diet (38%).

It has been established that 43% of the urban population and 63% of the rural population consume fish that was caught in water reservoirs of the Vologda Region. At the same time, the relationship between the content of mercury in hair and the consumption of local fish both in the city and in the countryside was statistically proven (U-Mann-Whitney test, U = -5.41 and U = 3.94, respectively; p = 0.000). The most common species consumed were perch (Perca fluviatilis), pike (Esox lucius), bream (Abramis brama), pike perch (Sander lucioperca) - representatives of predatory fish species.

**Figure 1:**
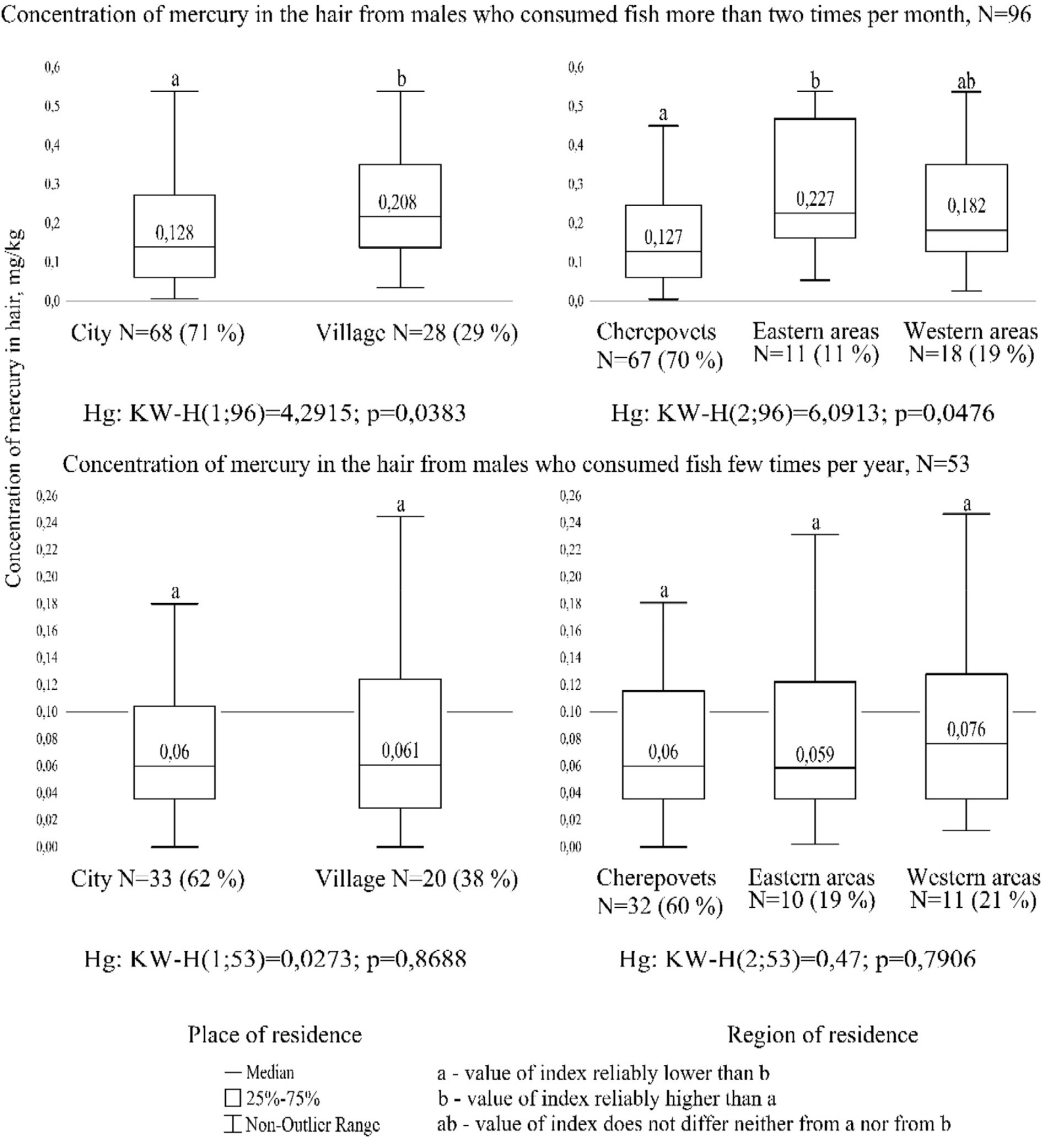
The mercury content in the hair of males aged 17–21 years living in the Vologda Region, depending on the frequency of consumption of fish, region and place of residence.

Among males who consume fish more than twice a month (Figure 1), statistically significant differences were found between the rural and urban populations (Mann-Whitney U, U = 2.07, p = 0.038), as well as between young men living in the city of Cherepovets and in the eastern areas of the region (Kruskal-Wallis, H = 6.09, df = 2, p = 0.048). In young people, in the diet of which fish is found several times a year, significant differences in places and regions of residence have not been established.

Wherein the median of the mercury content in the hair of males who consume fish more than two times per month is 3 times higher than the same indicators of males who consume fish less often in the urban population and 2 times higher than the same indicators of males who consume fish less often in the rural populations. Similarly, among representatives of different regions of residence: for males, in whose diet fish is often consumed, the content of heavy metal is more than 2 times higher than the same indicators of males who consume fish rarely.

**Figure 2:**
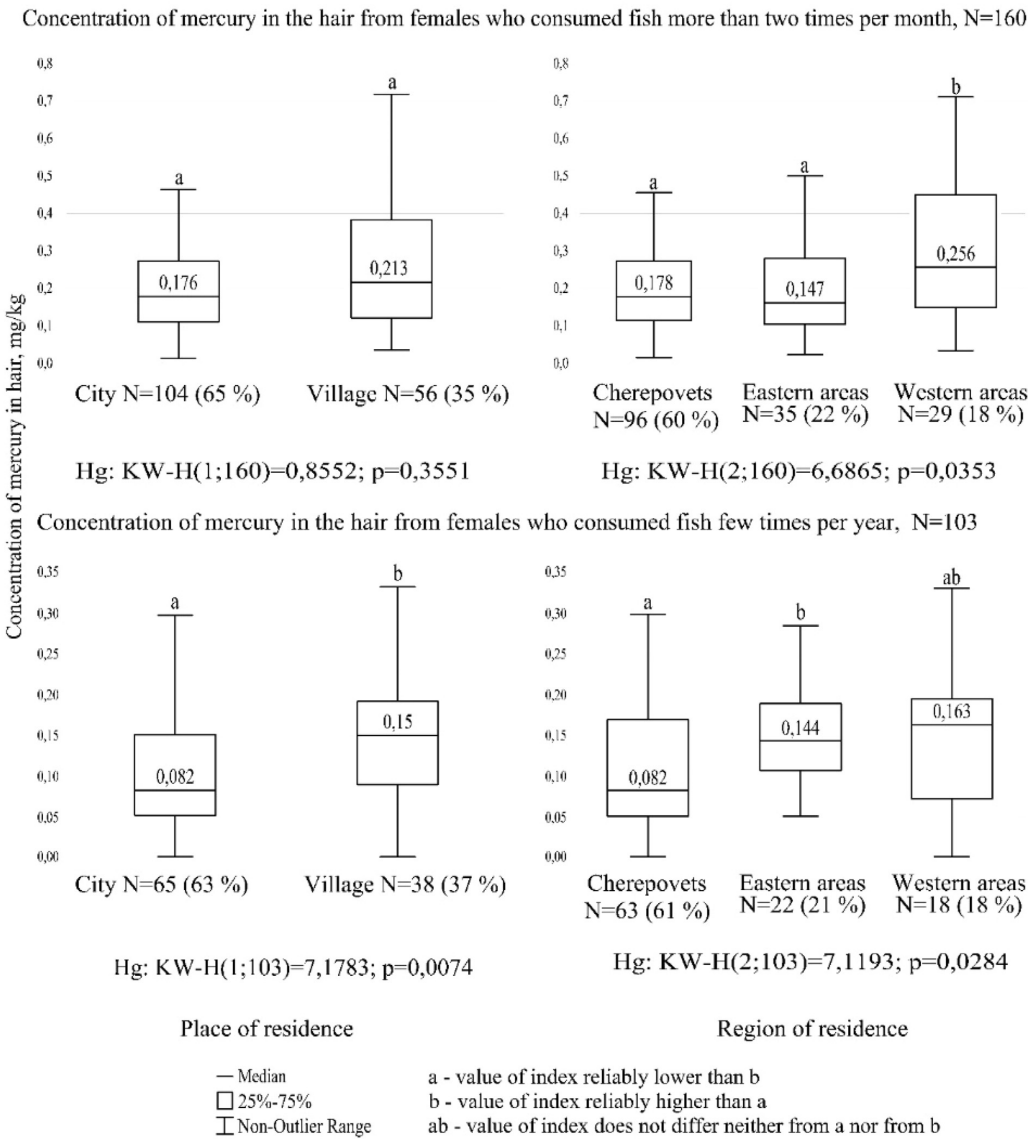
The mercury content in the hair of females aged 17–21 years living in the Vologda Region, depending on the frequency of consumption of fish, region and place of residence.

For females who consume fish more than 2 times a month (Figure 2), no statistically significant differences between the rural and urban populations were found (Mann-Whitney U, U = -0.92, p = 0.355). Among the study participants who practically excluded fish from the diet, the mercury content in the hair of people living in rural areas was significantly higher (Mann-Whitney U, U = 2.68, p = 0.007) than those living in urban environments. Nevertheless, among urban females who consume fish more than twice a month; the concentration of metal in their hair is 2 times that of those who consume fish several times a year.

Based on more frequent consumption of fish and regions of residence, representatives of the western areas have a metal concentration higher (Kruskal-Wallis, H = 6.609, df = 2, p = 0.035) than representatives of the city and the eastern areas of the region. Representatives of the eastern areas of the region with lesser fish consumption have statistically significant differences (Kruskal-Wallis, H = 7.12, df = 2, p = 0.028) with representatives of the city of Cherepovets.

However, for the entire sample, containing 50% of observations, the range of values of the attribute has a wider meaning for people who consume fish more than twice a month and for residents of the western areas of the region (except for males).

The revealed statistically significant differences suggest that there are cause-effect relationships between the mercury content in the hair of the residents of the Vologda Region aged 17–21 and the frequency of fish consumption, place and area of residence.

## 4 Discussion

The mercury concentration level in the hair for the entire sample is within the recommended values determined (2.0 mg/kg) by the World Health Organization (WHO) (WHO, 1990; NRC, 2000), moreover 95% of females are below the level (0.58 mg/kg), the excess of which may be accompanied by negative aspects of prenatal development (Lee et al., 2017).

The results are comparable with published data (median 0.164 mg/kg) from a study of a group of students (the chemistry department of the Wroclaw University of Technology) aged 21–22 living in an urban area of Poland (Chojnacka et al., 2010). At the same time, the established levels of mercury concentrations in the hair of students at ChSU is lower about half than those recorded in residents of the Kirillovsky district of the region under the age of 27 years (median 0.275 mg/kg) (Rumiantseva et al., 2018). The difference in the mercury content in the hair may be due to the amount of fish in the diet and its species composition.

So, in previous studies, it was recorded that the concentration of mercury in the hair positively correlates with the age of the subjects (Skalnaya et al., 2015; Gibb et al., 2016; Masih et al., 2016). According to researchers, this situation may be due to a significant decrease in liver and kidney function with age (Mangoni and Jackson, 2004), which in turn affects the excretion of heavy metal from the body (Skalnaya et al., 2015).

In the study sample, it was found that the concentration of mercury in the hair of females is higher than that of males. Similar results were obtained when comparing the values of the metal concentration between male (median 0.222 mg/kg for 10–19 years old and 0.241 mg/kg for 20–29 years old) and female (median 0.261 mg/kg for 10–19 years old and 0.298 mg/kg for 20–29 years) of the Orenburg Region. Also, in the study in question, it was found that gender differences in the levels of trace elements, including mercury, were less pronounced with age (Skalnaya et al., 2015). The opposite trend was observed when studying the mercury content in the hair of Polish students - in males (mean 0.216 mg/kg), the metal concentration is higher than in females (mean 0.204 mg/kg) (Chojnacka et al., 2010). However, statistically significant differences in the mercury content in the hair were noted only in this study.

It is likely that the higher mercury content in females' hairs compared to males may be due to nutritional preferences. Or, the sample studied was not equally distributed by gender, since most of the participants were females. However, some studies have shown that gender is not an important factor in determining the accumulation of mercury in hair (Yasutake et al., 2003; Agusa et al., 2005).

The analysis revealed statistically significant differences in the places of residence of the subjects. So, in the rural population, the metal content in the hair exceeds the indicators of city residents. Similar data are presented in a study in four states of the Malaysian Peninsula, in which the minimum concentration of mercury in the hair of the rural population is 1.5 times higher than the urban population (Hajeb et al., 2008). The authors explain these differences by the difference in diet, lifestyle and environmental factors.

A significant difference in the concentration of metal between groups of populations may be due to the specifics of the fish consumed. So in an urban environment, fish consumption includes: frozen and chilled fish (including those purchased at local markets), canned fish and preserves, as well as seafood; in rural areas, the population consumes fresh fish caught near their homes.

In overall, natural and geographical conditions of living on the territory of Vologda Region differ. According to the relief structure, the area can be described as a hilly plain: the central part and the east of the region are characterized by a hilly river landscape where large river basins are formed, the southwestern part of the region is a swampy vast lowland, and up to 92% of all lakes are concentrated in the northwestern part (Antipov, 1981; Vorobiev, 2007; Semenov and Troshichev, 2014). It is obvious that there are conditions for bioaccumulation and biomagnification of mercury through food chains in the western part of the region.

In particular, over the past decades, high concentrations of mercury in fish muscles have been recorded both from small lakes and from reservoirs of fishery importance in the western regions of the Vologda Region (Haines et al., 1995; 1995; Komov et al., 2004).

Thus higher levels of mercury in the hair of representatives of the western areas of the region, compared with residents of the eastern areas of the region and the cities, can be explained by the consumption of fish with a high metal content, that inhabits local water reservoirs (Komov et al., 2004).

The position of the consumed fish in the trophic chain also has a significant effect on the amount of mercury in human hair (Kidd et al., 2012; Bełdowska and Falkowska, 2016). According to the survey, the most common fish species consumed in food were representatives of predatory species.

Currently, most researchers conclude that fish consumption is a significant factor in the accumulation of heavy metal in the human body (Ha et al., 2017; Sheehan et al., 2014; Hajeb et al., 2008; Masih et al., 2016; Pinheiro et al., 2005). For our sample - students of the first year of study at ChSU - higher levels of metal concentration in the hair are observed in individuals whose diets have fish intake more than 2–3 times a month, which is consistent with the results of previous studies in Germany (Pesch et al., 2002), India (Masih et al., 2016) and Ethiopia (Habiba et al., 2017) according to which, the level of heavy metal in the hair rises with increasing frequency of fish consumption.

## 5 Conclusions

This study showed that the mercury content in the hair of students of ChSU of the first year of study is in a relatively wide range of values: from levels below the ability to determine metal with a device (less than 0.002 mg/kg) and does not exceed 1.7 mg/kg. The median values of the metal concentration in the hair of the examined students are close to those previously established for the contingent of young people in Eastern Europe and Russia, but lower than in Asia and Africa, where fish is a regular mandatory component of the diet of some population groups.

Higher levels of metal concentration in the hair of the rural population were recorded compared to urban, which can be explained by the consumption of fish with a high mercury content. Similar results have been repeatedly reported in the scientific literature. With a more detailed study, significant differences in the place of residence were found for the entire sample as a whole and for females. Also, gender differences are determined by a higher accumulation of mercury in females' hair.

Statistically significant differences were found in the content of mercury in the hair of females and males who consume fish more than twice a month in the regions of residence. The greatest difference in indicators (more than 2 times) was noted among females. The highest indices were observed among females - representatives of the western areas, among males - representatives of the eastern areas of the Vologda Region.

Thus, the frequency of fish consumption and region of residence can be a predictor of the content of mercury in the hair of young residents of the Vologda Region.

## Declarations

### Author contribution statement

Iuliia Aleksina: Conceived and designed the experiments; Analyzed and interpreted the data; Contributed reagents, materials, analysis tools or data; Wrote the paper.

Viktor Komov: Conceived and designed the experiments; Wrote the paper.

### Funding statement

This research did not receive any specific grant from funding agencies in the public, commercial, or not-for-profit sectors.

### Competing interest statement

The authors declare no conflict of interest.

### Additional information

No additional information is available for this paper.
