# Supplementary material for: Factors affecting the mercury concentration in the hair of young residents of the Vologda region, Russia
Source: Heliyon. 2020 Aug 5;6(8):e04580. doi: 10.1016/j.heliyon.2020.e04580 (PMC7413987; doi:10.1016/j.heliyon.2020.e04580)
Supplement: Supplementary Material [file mmc1.pdf]

Шифр	Концентра	Пол (м/ж)	Значение_	Возраст (п	Рост (см)	Вес (кг)	индекс Кетле		Место рож	Город1+Се	Место про	32_В3_Ч1	6. Какие п	Значение 6	9. Употреб	Значение 9	(1_2)_3_4_	10. Употре	Значение 1	11. Если да	Значение 1	Водоем
А040	0,2955	м	1	17	170	62	21,45329	2	ВО, Кадуйс	2	ВО, г. Чере	2	Мясные и	12	Два-три ра	4	4	Да	1	Нет ответа	0	Нет ответа
А034	0,0596	м	1	18	170	60	20,76125	2	ВО, г. Чере	1	ВО, г. Чере	1	Мясные и	1	Несколько	5	5	Нет	2	Нет ответа	0	Нет ответа
А032	0,0001	м	1	18	175	69	22,53061	2	ВО, г. Чере	1	ВО, г. Чере	1	Мясные и	10	Несколько	5	5	Нет	2	Семга, гор	4	Нет ответа
А036	1,677	м	1	18	Нет ответа	Нет ответа			ВО, г. Чере	1	ВО, г. Чере	1	Мясные и	15	Один раз в	3	3	Нет	2	Нет ответа	0	Нет ответа
А029	0,2514	ж	2	18	162	52	19,81405	2	ВО, г. Чере	1	ВО, г. Чере	1	Мясные и	8	Два-три ра	4	4	Да	1	Нет ответа	0	Нет ответа
А039	0,06	м	1	18	199	Нет ответа			ВО, г. Чере	1	ВО, г. Чере	1	Мясные и	1	Несколько	5	5	Нет	2	Нет ответа	0	Нет ответа
А030	0,0002	ж	2	19	165	55	20,20202	2	ВО, Шексн	2	ВО, г. Чере	2	Мясные и	7	Несколько	5	5	Нет	2	Нет ответа	0	Нет ответа
А035	0,0467	м	1	18	184	76	22,44802	2	ВО, г. Чере	1	ВО, г. Чере	1	Растительн	4	Два-три ра	4	4	Да	1	Мойва	5	Рыбинское
А037	0,1823	м	1	20	179	80	24,96801	2	ВО, г. Чере	1	ВО, г. Чере	1	Мясные и	1	Один раз в	3	3	Нет	2	Нет ответа	0	Нет ответа
А031	0,0786	м	1	18	175	55	17,95918	1	Республик	1	ВО, г. Чере	1	Мясные и	1	Два-три ра	4	4	Нет	2	Нет ответа	0	Нет ответа
А033	0,1756	м	1	18	173	64	21,38394	2	ВО, г. Чере	1	ВО, г. Чере	1	Мясные и	1	Два-три ра	4	4	Да	1	Нет ответа	0	Рыбинское
А041	0,1908	м	1	17	191	97	26,58918	3	ВО, г. Чере	1	ВО, г. Чере	1	Мясные и	1	Два-три ра	4	4	Нет	2	Нет ответа	0	Нет ответа
А075	0,072	м	1	18	173	81,2	27,13088	3	ВО, г. Чере	1	ВО, г. Чере	1	Мясные и	1	Несколько	5	5	Нет	2	Нет ответа	0	Нет ответа
А077	0,4303	м	1	18	181	80	24,41928	2	ВО, г. Чере	1	ВО, г. Чере	1	Мясные и	1	Два-три ра	4	4	Нет	2	Нет ответа	0	Нет ответа
А078	0,0534	м	1	19	175	60	19,59184	2	ВО, г. Чере	1	ВО, г. Чере	1	Мясные и	1	Два-три ра	4	4	Нет	2	Нет ответа	0	Нет ответа
А081	0,0592	м	1	19	178	77	24,30249	2	ВО, Никол	2	ВО, г. Чере	3	Мясные и	12	Несколько	5	5	Да	1	Хариуз	9	Нет ответа
А084	0,2429	м	1	18	173	75	25,05931	3	ВО, г. Чере	1	ВО, г. Чере	1	Мясные и	8	Несколько	5	5	Да	1	Нет ответа	0	Нет ответа
А083	0,2794	ж	2	18	169	61	21,3578	2	ВО, Сокол	2	ВО, г. Чере	3	Мясные и	1	Несколько	5	5	Да	1	Окунь	7	оз. Кубенск
А079	0,4695	м	1	18	189	90	25,19526	3	ВО, г. Чере	1	ВО, г. Чере	1	Мясные и	20	Не употреб	6	6	Нет	2	Нет ответа	0	Нет ответа
А073	0,3853	ж	2	18	170	68	23,52941	2	ВО, г. Чере	1	ВО, г. Чере	1	Мясные и	7	Два-три ра	4	4	Нет	2	Нет ответа	0	Нет ответа
А074	0,2213	ж	2	18	162	57	21,71925	2	ВО, г. Чере	1	ВО, г. Чере	1	Растительн	13	Два-три ра	4	4	Да	1	Нет ответа	0	р. Шексна
А080	0,3525	м	1	18	173	69	23,05456	2	ВО, г. Чере	1	ВО, г. Чере	1	Мясные и	7	Один раз в	3	3	Да	1	Нет ответа	0	Рыбинское
А082	0,1063	ж	2	18	162	62	23,62445	2	ВО, г. Чере	1	ВО, г. Чере	1	Мясные и	20	Два-три ра	4	4	Нет	2	Нет ответа	0	Нет ответа
А076	0,1288	м	1	19	174	66	21,79945	2	ВО, г. Чере	1	ВО, г. Чере	1	Мясные и	20	Два-три ра	4	4	Да	1	Щука	1	Нет ответа
А050	0,2467	м	1	18	179	63	19,66231	2	Челябинск	2	ВО, Черепо	2	Мясные и	1	Несколько	5	5	Да	1	Щука, суда	6	Река
А068	0,211	м	1	19	174	Нет ответа			ВО, Вожег	2	ВО, г. Чере	2	Мясные и	1	Один раз в	3	3	Да	1	Щука, окун	3	Река
А065	0,2882	м	1	19	177	60	19,15158	2	ВО, г. Чере	1	ВО, г. Чере	1	Мясные и	1	Два-три ра	4	4	Да	1	Щука, окун	3	Рыбинское
А067	0,0762	м	1	18	171	Нет ответа			ВО, г. Чере	1	ВО, г. Чере	1	Мясные и	1	Несколько	5	5	Нет	2	Нет ответа	0	Нет ответа
А066	0,5368	м	1	18	172	68	22,9854	2	ВО, Кирил	2	ВО, г. Чере	2	Мясные и	20	Два-три ра	4	4	Да	1	Нет ответа	0	Нет ответа
А070	0,0921	м	1	18	181	57	17,39874	1	ВО, г. Чере	1	ВО, г. Чере	1	Мясные и	20	Один раз в	3	3	Нет	2	Нет ответа	0	Нет ответа
А069	0,0504	м	1	18	180	63	19,44444	2	ВО, г. Чере	1	ВО, г. Чере	1	Мясные и	20	Два-три ра	4	4	Нет	2	Нет ответа	0	Нет ответа
А049	0,1783	м	1	18	173	51	17,04033	1	ВО, г. Чере	1	ВО, г. Чере	1	Мясные и	12	Несколько	5	5	Да	1	Нет ответа	0	Нет ответа
А004	0,7344	ж	2	18	164	Нет ответа			ВО, г. Чере	1	ВО, г. Чере	1	Мясные и	9	Один раз в	3	3	Нет	2	Нет ответа	0	Нет ответа
А007	0,1518	м	1	18	176	74	23,88946	2	ВО, Нюксе	2	ВО, г. Чере	3	Мясные и	7	Два-три ра	4	4	Да	1	Щука	1	Река
А001	0,0764	м	1	18	179	65	20,28651	2	ВО, Бабаев	2	ВО, г. Чере	2	Хлебобулс	6	Несколько	5	5	Да	1	Нет ответа	0	Нет ответа
А003	0,1997	ж	2	18	168	60	21,2585	2	ВО, г. Чере	1	ВО, г. Чере	1	Мясные и	8	Два-три ра	4	4	Нет	2	Нет ответа	0	Нет ответа
А005	0,0842	ж	2	17	164	47	17,47472	1	ВО, Бабаев	2	ВО, г. Чере	2	Хлебобулс	3	Несколько	5	5	Да	1	Нет ответа	0	Нет ответа
А008	0,4414	м	1	20	175	75	24,4898	2	ВО, г. Чере	1	ВО, г. Чере	1	Мясные и	1	Два-три ра	4	4	Да	1	Щука, суда	2	р. Шексна
А002	0,0138	ж	2	18	161	45	17,36044	1	ВО, г. Чере	1	ВО, г. Чере	1	Мясные и	7	Два-три ра	4	4	Да	1	Нет ответа	0	Нет ответа
А021	0,1206	ж	2	18	169	Нет ответа			ВО, Сокол	2	ВО, г. Чере	3	Мясные и	7	Не употреб	6	6	Нет	2	Нет ответа	0	Нет ответа
А017	0,0728	м	1	17	182	57	17,20807	1	Волгоград	1	ВО, г. Чере	1	Мясные и	12	Несколько	5	5	Нет	2	Нет ответа	0	Нет ответа
А018	0,0465	м	1	18	168	51	18,06973	1	ВО, г. Чере	1	ВО, г. Чере	1	Мясные и	1	Два-три ра	4	4	Нет	2	Нет ответа	0	Нет ответа
А025	0,2018	м	1	18	183	81	24,18705	2	ВО, г. Чере	1	ВО, г. Чере	1	Мясные и	7	Два-три ра	4	4	Нет	2	Нет ответа	0	Нет ответа
А020	0,062	ж	2	18	158	54	21,63115	2	ВО, г. Чере	1	ВО, г. Чере	1	Курица	14	Несколько	5	5	Нет	2	Нет ответа	0	Нет ответа
А024	0,0417	м	1	18	182	90	27,17063	3	ВО, г. Чере	1	ВО, г. Чере	1	Растительн	4	Несколько	5	5	Нет	2	Нет ответа	0	Нет ответа
А027	0,3103	ж	2	17	162	58	22,10029	2	ВО, г. Чере	1	ВО, г. Чере	1	Мясные и	1	Два-три ра	4	4	Нет	2	Нет ответа	0	Нет ответа
А019	0,0643	м	1	18	182	73	22,0384	2	ВО, Верхов	2	ВО, г. Чере	3	Мясные и	8	Несколько	5	5	Да	1	Щука, окун	3	Река
А028	0,201	ж	2	17	164	50	18,59012	2	ВО, Грязов	2	ВО, г. Чере	3	Хлебобулс	3	Два-три ра	4	4	Да	1	Щука, окун	3	р. Сухона
А026	0,5376	ж	2	18	164	59	21,93635	2	ВО, г. Чере	1	ВО, г. Чере	1	Мясные и	1	Два-три ра	2	12	Нет	2	Нет ответа	0	Нет ответа
А022	0,2115	ж	2	18	173	55	18,37683	1	ВО, г. Чере	1	ВО, г. Чере	1	Мясные и	12	Два-три ра	4	4	Нет	2	Щука	1	Река
А064	0,0789	ж	2	18	165	51	18,73278	2	ВО, г. Чере	1	ВО, г. Чере	1	Хлебобулс	3	Не употреб	6	6	Нет	2	Нет ответа	0	Нет ответа
А054	0,4826	ж	2	19	157	50	20,2848	2	ВО, г. Чере	1	ВО, г. Чере	1	Мясные и	1	Несколько	5	5	Да	1	Щука, суда	6	Рыбинское
А057	0,1918	м	1	18	178	69	21,77755	2	ВО, Черепо	2	ВО, Черепо	2	Мясные и	1	Несколько	5	5	Да	1	Нет ответа	0	Нет ответа

A051	0,0001	ж	2	18	168	53	18,77834	2	ВО, г. Чере	1	ВО, г. Чере	1	Мясные и	19	Не употреб	6	6	Нет	2	Нет ответа	0	Нет ответа
A059	0,1972	ж	2	18	164	62	23,05175	2	ВО, г. Чере	1	ВО, г. Чере	1	Мясные и	12	Два-три ра	4	4	Нет	2	Нет ответа	0	Нет ответа
A063	0,2687	ж	2	18	165	57	20,93664	2	ВО, г. Чере	1	ВО, г. Чере	1	Хлебобуло	3	Два-три ра	4	4	Нет	2	Нет ответа	0	Нет ответа
A058	0,1027	ж	2	18	159	58	22,94213	2	ВО, г. Чере	1	ВО, г. Чере	1	Мясные и	12	Два-три ра	4	4	Нет	2	Нет ответа	0	Нет ответа
A053	0,1449	ж	2	18	176	76	24,53512	2	ВО, Тарног	2	ВО, г. Чере	3	Мясные и	19	Несколько	5	5	Да	1	Нет ответа	0	Нет ответа
A060	0,0157	м	1	18	185	Нет ответа			ВО, Тотем	2	ВО, г. Чере	3	Мясные и	1	Несколько	5	5	Нет	2	Нет ответа	0	Нет ответа
A056	0,0895	ж	2	18	166	44	15,96748	1	ВО, г. Чере	1	ВО, г. Чере	1	Мясные и	19	Несколько	5	5	Нет	2	Нет ответа	0	Нет ответа
A052	0,0408	ж	2	18	163	60	22,58271	2	ВО, Белозе	2	ВО, г. Чере	2	Мясные и	19	Не употреб	6	6	Нет	2	Нет ответа	0	Нет ответа
A061	0,1447	ж	2	18	170	60	20,76125	2	ВО, г. Чере	1	ВО, г. Чере	1	Хлебобуло	21	Несколько	5	5	Нет	2	Нет ответа	0	Нет ответа
A044	0,4676	ж	2	18	167	50	17,92822	1	ВО, Никол	2	ВО, г. Чере	3	Хлебобуло	17	Два-три ра	4	4	Да	1	Щука	1	р. Кема, р.
A043	0,0809	м	1	19	170	63	21,79931	2	ВО, г. Чере	1	ВО, г. Чере	1	Мясные и	16	Два-три ра	2	12	Да	1	Нет ответа	0	Нет ответа
A048	0,1149	м	1	18	176	67	21,62965	2	ВО, Вожег	2	ВО, г. Чере	2	Крупы	11	Два-три ра	4	4	Да	1	Окунь, щу	8	оз. Воже
A046	0,2463	м	1	18	182	78	23,54788	2	ВО, Кирил	2	ВО, г. Чере	2	Хлебобуло	18	Два-три ра	4	4	Да	1	Окунь	7	оз. Сиверс
A047	0,0436	м	1	18	180	64	19,75309	2	ВО, Череп	2	ВО, Череп	2	Мясные и	1	Два-три ра	4	4	Да	1	Нет ответа	0	Нет ответа
A042	0,0588	м	1	18	173	72	24,05693	2	ВО, г. Чере	1	ВО, г. Чере	1	Хлебобуло	3	Несколько	5	5	Нет	2	Нет ответа	0	Нет ответа
A045	0,4839	ж	2	18	164	54	20,07733	2	ВО, Вожег	2	ВО, г. Чере	2	Мясные и	1	Несколько	5	5	Да	1	Щука, суда	6	оз. Воже
A009	0,101	ж	2	18	166	51	18,50777	2	ВО, г. Чере	1	ВО, г. Чере	1	Растительн	4	Один раз в	3	3	Нет	2	Нет ответа	0	Нет ответа
A016	0,1787	ж	2	18	158	45	18,02596	1	ВО, г. Чере	1	ВО, г. Чере	1	Растительн	13	Один раз в	3	3	Нет	2	Нет ответа	0	Нет ответа
A011	0,058	м	1	18	183	80	23,88844	2	ВО, г. Чере	1	ВО, г. Чере	1	Мясные и	1	Один раз в	3	3	Нет	2	Нет ответа	0	Нет ответа
A014	0,3453	м	1	18	176	Нет ответа			ВО, Кадуй	2	ВО, г. Чере	2	Крупы	11	Один раз в	3	3	Да	1	Нет ответа	0	Нет ответа
A015	0,0519	м	1	19	179	79	24,65591	2	ВО, г. Чере	1	ВО, г. Чере	1	Мясные и	12	Несколько	5	5	да	1	Нет ответа	0	р. Шексна
A012	0,1922	ж	2	17	168	58	20,54989	2	ВО, г. Чере	1	ВО, г. Чере	1	Мясные и	7	Несколько	5	5	Нет	2	Нет ответа	0	Нет ответа
A010	0,0647	м	1	18	174	68	22,46003	2	ВО, г. Чере	1	ВО, г. Чере	1	Мясные и	1	Один раз в	3	3	Да	1	Нет ответа	0	Нет ответа
A164	0,0281	ж	2	18	166	56	20,32225	2	ВО, г. Чере	1	ВО, г. Чере	1	Мясные и	7	Два-три ра	4	4	Да	1	Щука, оку	3	Вашкински
A166	0,1589	ж	2	18	157	56	22,71897	2	ВО, г. Чере	1	ВО, г. Чере	1	Рыба и ры	26	Практичес	1	12	Нет	2	Нет ответа	0	Нет ответа
A167	0,3055	м	1	18	181	Нет ответа			ВО, г. Чере	1	ВО, г. Чере	1	Рыба и ры	26	Один раз в	3	3	Да	1	Окунь	7	Нет ответа
A152	0,0597	м	1	18	184	99	29,24149	3	Краснодак	1	ВО, г. Чере	1	Мясные и	1	Не употреб	6	6	Нет	2	Нет ответа	0	Нет ответа
A159	0,08	ж	2	18	170	58	20,0692	2	ВО, г. Чере	1	ВО, г. Чере	1	Молочная	22	Два-три ра	4	4	Нет	2	Нет ответа	0	Нет ответа
A160	0,0814	ж	2	18	168	51	18,06973	1	ВО, г. Чере	1	ВО, г. Чере	1	Мясные и	7	Не употреб	6	6	Нет	2	Нет ответа	0	Нет ответа
A153	0,3774	ж	2	18	168	59	20,9042	2	ВО, г. Чере	1	ВО, г. Чере	1	Мясные и	7	Один раз в	3	3	Да	1	Лещ, суда	15	Нет ответа
A154	0,1007	м	1	18	168	69	24,44728	2	ВО, Кадуй	2	ВО, г. Чере	2	Мясные и	7	Два-три ра	4	4	Да	1	Нет ответа	0	Нет ответа
A165	0,1565	ж	2	18	162	50	19,05197	2	ВО, г. Чере	1	ВО, г. Чере	1	Мясные и	29	Два-три ра	4	4	Нет	2	Нет ответа	0	Нет ответа
A163	0,0024	м	1	17	172	72	24,33748	2	ВО, Грязов	2	ВО, г. Чере	3	Мясные и	8	Не употреб	6	6	Нет	2	Нет ответа	0	Нет ответа
A150	0,1576	ж	2	18	174	64	21,13886	2	ВО, Кадуй	2	ВО, г. Чере	2	Мясные и	29	Несколько	5	5	Да	1	Нет ответа	0	р. Суда
A157	0,1268	м	1	18	180	68	20,98765	2	ВО, Кадуй	2	ВО, г. Чере	2	Мясные и	19	Несколько	5	5	Да	1	Нет ответа	0	р. Шексна
A151	0,0578	ж	2	18	Нет ответа	Нет ответа			ВО, г. Чере	1	ВО, г. Чере	1	Мясные и	1	Несколько	5	5	Нет	2	Нет ответа	0	Нет ответа
A161	0,1905	ж	2	18	168	60	21,2585	2	ВО, г. Чере	1	ВО, г. Чере	1	Мясные и	29	Несколько	5	5	Да	1	Нет ответа	0	Нет ответа
A149	0,0592	м	1	19	175	Нет ответа			ВО, Волого	2	ВО, г. Чере	3	Мясные и	20	Несколько	5	5	Да	1	Нет ответа	0	р. Шексна,
A155	0,1864	ж	2	18	160	55	21,48438	2	ВО, г. Чере	1	ВО, г. Чере	1	Мясные и	16	Два-три ра	4	4	Нет	2	Нет ответа	0	Нет ответа
A158	0,3571	ж	2	18	155	42	17,48179	1	ВО, Шексн	2	ВО, г. Чере	2	Мясные и	19	Два-три ра	4	4	Нет	2	Нет ответа	0	Нет ответа
A162	0,1607	ж	2	17	166	60	21,77384	2	ВО, г. Волс	1	ВО, г. Чере	3	Хлебобуло	6	Два-три ра	4	4	Нет	2	Нет ответа	0	Нет ответа
A156	0,2087	ж	2	18	156	45	18,49112	2	ВО, Усть-К	2	ВО, г. Чере	2	Мясные и	1	Не употреб	6	6	Нет	2	Нет ответа	0	Нет ответа
A119	0,2082	ж	2	18	167	56	20,0796	2	ВО, г. Чере	1	ВО, г. Чере	1	Мясные и	16	Один раз в	3	3	Да	1	Нет ответа	0	Нет ответа
A116	0,7636	м	1	18	175	70	22,85714	2	ВО, г. Чере	1	ВО, г. Чере	1	Растительн	4	Два-три ра	4	4	Нет	2	Нет ответа	0	Нет ответа
A124	0,044	ж	2	18	160	49	19,14063	2	ВО, г. Чере	1	ВО, г. Чере	1	Хлебобуло	3	Не употреб	6	6	Нет	2	Нет ответа	0	Нет ответа
A123	0,0974	ж	2	17	165	53	19,4674	2	ВО, Грязов	2	ВО, г. Чере	3	Хлебобуло	3	Два-три ра	4	4	Нет	2	Нет ответа	0	Нет ответа
A122	0,1404	м	1	18	164	65	24,16716	2	ВО, г. Чере	1	ВО, г. Чере	1	Мясные и	1	Два-три ра	4	4	Нет	2	Нет ответа	0	Нет ответа
A125	0,0082	ж	2	18	163	58	21,82995	2	ВО, г. Чере	1	ВО, г. Чере	1	Мясные и	19	Не употреб	6	6	Нет	2	Нет ответа	0	Нет ответа
A127	0,1132	ж	2	18	166	55	19,95936	2	ВО, г. Чере	1	ВО, г. Чере	1	Мясные и	8	Два-три ра	4	4	Нет	2	Нет ответа	0	Нет ответа
A115	0,1717	ж	2	18	156	46	18,90204	2	ВО, г. Чере	1	ВО, г. Чере	1	Мясные и	8	Несколько	5	5	Нет	2	Нет ответа	0	Нет ответа
A120	0,4483	ж	2	18	172	60	20,28123	2	ВО, Усть-К	2	ВО, г. Чере	2	Мясные и	1	Два-три ра	4	4	Да	1	Щука	1	оз. Кубенс
A118	0,3585	ж	2	18	165	48	17,63085	1	ВО, г. Чере	1	ВО, г. Чере	1	Мясные и	8	Несколько	5	5	Да	1	Нет ответа	0	Нет ответа
A121	0,1656	ж	2	19	165	70	25,71166	3	ВО, г. Чере	1	ВО, г. Чере	1	Рыба и ры	26	Один раз в	3	3	Нет	2	Нет ответа	0	Нет ответа
A201	0,035	ж	2	19	167	60	21,51386	2	ВО, г. Волс	1	ВО, г. Чере	3	Рыба и ры	2	Практичес	1	12	Нет	2	Нет ответа	0	Нет ответа

A202	0,0132	м	1	18	180	58	17,90123	4	ВО, г. Чере	1	ВО, г. Чере	1	Мясные и	28	Не употреб	6	6	Нет	2	Нет ответа	0	Нет ответа
A207	0,1401	ж	2	18	157	50	20,2848	2	ВО, г. Чере	1	ВО, г. Чере	1	Мясные и	1	Два-три ра	2	12	Да	1	Нет ответа	0	Нет ответа
A206	0,0553	ж	2	18	152	49	21,20845	2	ВО, Волого	2	ВО, г. Чере	3	Хлебобуло	6	Два-три ра	4	4	Нет	2	Нет ответа	0	Нет ответа
A203	0,0388	ж	2	18	167	53	19,00391	2	ВО, г. Чере	1	ВО, г. Чере	1	Мясные и	7	Несколько	5	5	Нет	2	Нет ответа	0	Нет ответа
A204	0,141	ж	2	18	170	53	18,3391	1	ВО, г. Чере	1	ВО, г. Чере	1	Раститель	4	Один раз в	3	3	Нет	2	Нет ответа	0	Нет ответа
A200	0,0521	ж	2	18	153	55	23,49524	2	ВО, г. Чере	1	ВО, г. Чере	1	Молочная	22	Несколько	5	5	Да	1	Нет ответа	0	Нет ответа
A205	0,1276	м	1	21	173	51	17,04033	1	ВО, г. Чере	1	ВО, г. Чере	1	Нет ответа	0	Несколько	5	5	Нет	2	Нет ответа	0	Нет ответа
A199	0,2243	ж	2	17	165	75	27,54821	3	ВО, г. Чере	1	ВО, г. Чере	1	Мясные и	2	Два-три ра	2	12	Да	1	Нет ответа	0	Рыбинское
A101	0,3826	ж	2	18	167	80	28,68514	3	ВО, г. Чере	1	ВО, г. Чере	1	Хлебобуло	3	Два-три ра	4	4	Да	1	Нет ответа	0	р. Шексна
A112	0,0611	м	1	18	179	78	24,34381	2	ВО, г. Чере	1	ВО, г. Чере	1	Мясные и	1	Два-три ра	4	4	Да	1	Щука, окун	3	Нет ответа
A106	0,0187	ж	2	18	164	51	18,96193	2	ВО, г. Чере	1	ВО, г. Чере	1	Раститель	4	Не употреб	6	6	Нет	2	Нет ответа	0	Нет ответа
A109	0,1852	ж	2	18	154	56	23,61275	2	ВО, Тотем	2	ВО, г. Чере	3	Крупы; суп	24	Не употреб	6	6	Нет	2	Нет ответа	0	Нет ответа
A100	0,0078	м	1	18	172	59	19,94321	2	ВО, г. Чере	1	ВО, г. Чере	1	Мясные и	20	Практичес	1	12	Да	1	Нет ответа	0	Рыбинское
A110	0,0364	ж	2	17	153	54	23,06805	2	ВО, Волого	2	ВО, г. Чере	3	Мясные и	20	Два-три ра	4	4	Да	1	Нет ответа	0	Нет ответа
A104	0,0936	ж	2	18	160	49	19,14063	2	ВО, г. Чере	1	ВО, г. Чере	1	Раститель	4	Несколько	5	5	Нет	2	Нет ответа	0	Нет ответа
A113	0,046	ж	2	18	166	54	19,59646	2	ВО, г. Чере	1	ВО, г. Чере	1	Мясные и	20	Несколько	5	5	Нет	2	Нет ответа	0	Нет ответа
A108	0,0219	ж	2	18	173	57	19,04507	2	ВО, Сокол	1	ВО, г. Чере	1	Раститель	4	Несколько	5	5	Нет	2	Нет ответа	0	Нет ответа
A103	0,3798	ж	2	20	164	52	19,33373	2	ВО, Череп	2	ВО, Череп	2	Мясные и	1	Один раз в	3	3	Да	1	Щука, лещ	11	Нет ответа
A111	0,048	м	1	18	180	79	24,38272	2	ВО, г. Чере	1	ВО, г. Чере	1	Мясные и	25	Несколько	5	5	Нет	2	Нет ответа	0	Нет ответа
A107	0,2899	ж	2	18	156	56	23,01118	2	ВО, г. Чере	1	ВО, г. Чере	1	Мясные и	12	Два-три ра	4	4	Нет	2	Нет ответа	0	Нет ответа
A114	0,3313	ж	2	18	164	50	18,59012	2	ВО, Кадуй	2	ВО, г. Чере	2	Хлебобуло	6	Несколько	5	5	Да	1	щука	1	Нет ответа
A105	0,1114	ж	2	18	164	50	18,59012	2	ВО, г. Чере	1	ВО, г. Чере	1	Хлебобуло	3	Один раз в	3	3	Да	1	Нет ответа	0	р. Шексна
A183	0,2311	ж	2	18	159	53	20,96436	2	ВО, г. Чере	1	ВО, г. Чере	1	Рыба и ры	26	Практичес	1	12	Нет	2	Нет ответа	0	Нет ответа
A180	0,6705	ж	2	18	159	48	18,98659	2	ВО, г. Чере	1	ВО, г. Чере	1	Раститель	4	Один раз в	3	3	Да	1	Нет ответа	0	Нет ответа
A181	0,0556	ж	2	18	170	75	25,95156	3	ВО, г. Чере	1	ВО, г. Чере	1	Мясные и	16	Два-три ра	4	4	Нет	2	Нет ответа	0	Нет ответа
A185	0,1703	ж	2	18	160	60	23,4375	2	ВО, г. Чере	1	ВО, г. Чере	1	Рыба и ры	32	Несколько	5	5	Нет	2	Нет ответа	0	Нет ответа
A189	0,2235	ж	2	18	161	Нет ответа			ВО, г. Чере	1	ВО, г. Чере	1	Рыба и ры	26	Практичес	1	12	Нет	2	Нет ответа	0	Нет ответа
A179	0,1264	ж	2	18	160	70	27,34375	3	ВО, Никол	2	ВО, г. Чере	3	Мясные и	28	Два-три ра	2	12	Нет	2	Нет ответа	0	Нет ответа
A187	0,1364	ж	2	18	165	46	16,89624	1	ВО, г. Чере	1	ВО, г. Чере	1	Мясные и	20	Два-три ра	4	4	Да	1	Нет ответа	0	Нет ответа
A186	0,4113	ж	2	18	164	59	21,93635	2	ВО, г. Чере	1	ВО, г. Чере	1	Крупы; мо	33	Два-три ра	4	4	Да	1	Нет ответа	0	Нет ответа
A182	0,3755	ж	2	18	172	60	20,28123	2	ВО, Череп	2	ВО, г. Чере	2	Кондитерс	5	Один раз в	3	3	Да	1	Окунь	7	р. Суда
A135	1,028	м	1	17	180	65	20,06173	2	ВО, Бабае	2	ВО, г. Чере	2	Мясные и	1	Два-три ра	2	12	Да	1	Щука, окун	3	Нет ответа
A129	0,0942	м	1	18	172	67	22,64738	2	ВО, г. Чере	1	ВО, г. Чере	1	Рыба и ры	26	Два-три ра	2	12	Нет	2	Нет ответа	0	Нет ответа
A143	0,2686	ж	2	18	167	64	22,94812	2	ВО, г. Чере	1	ВО, г. Чере	1	Раститель	4	Два-три ра	4	4	Нет	2	Нет ответа	0	Нет ответа
A141	0,5386	м	1	18	177	80	25,53545	3	ВО, г. Волс	1	ВО, г. Чере	3	Мясные и	1	Один раз в	3	3	Нет	2	Нет ответа	0	Нет ответа
A134	0,0552	ж	2	18	156	43	17,6693	1	ВО, г. Чере	2	ВО, Череп	2	Раститель	4	Несколько	5	5	Нет	2	Нет ответа	0	Нет ответа
A137	0,1654	ж	2	18	164	55	20,44914	2	ВО, Сокол	2	ВО, г. Чере	3	Мясные и	20	Несколько	5	5	Да	1	Щука, окун	3	р. Вологда
A131	0,2881	ж	2	18	168	55	19,48696	2	ВО, г. Чере	1	ВО, г. Чере	1	Мясные и	7	Один раз в	3	3	Да	1	Нет ответа	0	Нет ответа
A132	0,0227	ж	2	17	158	57	22,83288	2	ВО, г. Чере	1	ВО, г. Чере	1	Мясные и	27	Два-три ра	4	4	Да	1	Нет ответа	0	Нет ответа
A136	0,0151	ж	2	18	164	52	19,33373	2	Архангель	1	ВО, г. Чере	1	Мясные и	28	Два-три ра	4	4	Нет	2	Нет ответа	0	Нет ответа
A128	1,195	ж	2	17	160	49	19,14063	2	ВО, Вытег	2	ВО, г. Чере	2	Хлебобуло	3	Два-три ра	4	4	Да	1	Щука, суда	2	оз. Ковжск
A142	0,0738	м	1	18	178	82	25,88057	3	ВО, г. Чере	1	ВО, г. Чере	1	Мясные и	0	Два-три ра	2	12	Нет	2	Нет ответа	0	Нет ответа
A139	0,1144	ж	2	18	160	46	17,96875	1	ВО, Усть-К	2	ВО, г. Чере	2	Мясные и	20	Один раз в	3	3	Да	1	Нет ответа	0	оз. Кубенс
A133	0,0736	ж	2	18	167	55	19,72104	2	ВО, г. Чере	1	ВО, г. Чере	1	Хлебобуло	6	Несколько	5	5	Да	1	Окунь	7	Рыбинское
A130	0,4134	ж	2	17	170	69	23,87543	2	ВО, Тотем	2	ВО, г. Чере	3	Мясные и	20	Несколько	5	5	Да	1	Щука, лещ	11	Нет ответа
A097	0,7901	м	1	18	175	75	24,4898	2	ВО, г. Чере	1	ВО, г. Чере	1	Мясные и	7	два-три ра	4	4	Да	1	Нет ответа	0	р. Шексна
A093	0,1597	ж	2	17	163	57	21,45357	2	ВО, г. Чере	1	ВО, г. Чере	1	Мясные и	8	Два-три ра	4	4	Да	1	Лещ, судак	15	Рыбинское
A089	0,1065	ж	2	19	170	55	19,03114	2	ВО, Волого	2	ВО, г. Чере	3	Раститель	4	Два-три ра	4	4	Нет	2	Нет ответа	0	Нет ответа
A088	0,1364	ж	2	18	172	68	22,9854	2	ВО, г. Чере	1	ВО, г. Чере	1	Мясные и	1	Несколько	5	5	Да	1	Судак, сом	12	Рыбинское
A098	0,0878	ж	2	18	154	57	24,03441	2	Армения,	1	ВО, г. Чере	1	Мясные и	23	Два-три ра	4	4	Нет	2	Нет ответа	0	Нет ответа
A091	0,3046	ж	2	17	161	Нет ответа			ВО, Никол	2	ВО, г. Чере	3	Раститель	4	Два-три ра	4	4	Да	1	Щука, кара	13	р. Юг
A090	0,0377	ж	2	17	163	50	18,81892	2	ВО, Белозе	2	ВО, г. Чере	2	Мясные и	8	Два-три ра	4	4	Да	1	Нет ответа	0	Нет ответа
A092	0,2166	ж	2	18	158	53	21,23057	2	ВО, г. Чере	1	ВО, г. Чере	1	Мясные и	20	Два-три ра	4	4	Да	1	Лещ	14	Нет ответа
A094	0,1458	ж	2	18	178	67	21,14632	2	ВО, Верхов	2	ВО, г. Чере	3	Хлебобуло	3	Два-три ра	2	12	Да	1	Нет ответа	0	Рыбинское

A087	0,6322	ж	2	18	165	Нет ответа			ВО, г. Чере	1	ВО, г. Чере	1	Растительн	4	Два-три ра	4	4	Да	1	Щука, леш	11	оз. Белое
A086	0,4912	м	1	17	190	80	22,16066	2	ВО, Кич-Го	2	ВО, г. Чере	3	Мясные и	1	Два-три ра	4	4	Да	1	Нет ответа	0	Нет ответа
A099	0,1247	ж	2	20	160	55	21,48438	2	ВО, г. Чере	1	ВО, г. Чере	1	Мясные и	23	Несколько	5	5	Нет	2	Нет ответа	0	Нет ответа
A095	0,0772	ж	2	20	159	50	19,7777	2	ВО, Волог	2	ВО, г. Чере	3	Мясные и	20	Несколько	5	5	Нет	2	Нет ответа	0	Нет ответа
A085	0,7132	м	1	21	184	61	18,01749	1	ВО, г. Чере	1	ВО, г. Чере	1	Мясные и	1	Несколько	5	5	Да	1	Судак	10	Рыбинское
A168	0,4387	м	1	18	180	55	16,97531	1	ВО, г. Чере	1	ВО, г. Чере	1	Рыба и ры	2	Два-три ра	2	12	Да	1	Нет ответа	0	Нет ответа
A169	0,0726	м	1	18	168	69	24,44728	2	ВО, г. Чере	1	ВО, г. Чере	1	Мясные и	19	Один раз в	3	3	Нет	2	Нет ответа	0	Нет ответа
A170	0,2406	ж	2	18	166	53	19,23356	2	ВО, г. Чере	1	ВО, г. Чере	1	Мясные и	8	Два-три ра	4	4	Нет	2	Нет ответа	0	Нет ответа
A178	0,4364	м	1	20	182	75	22,64219	2	ВО, г. Чере	1	ВО, г. Чере	1	Мясные и	1	Один раз в	3	3	Нет	2	Нет ответа	0	Нет ответа
A176	0,1312	ж	2	18	168	65	23,03005	2	ВО, г. Чере	1	ВО, г. Чере	1	Мясные и	7	Два-три ра	4	4	Нет	2	Нет ответа	0	Нет ответа
A171	0,2525	м	1	18	176	84	27,11777	3	Украина, Д	1	ВО, г. Чере	1	Хлебобуло	21	Несколько	5	5	Нет	2	Нет ответа	0	Нет ответа
A177	0,0404	м	1	18	180	75	23,14815	2	ВО, г. Чере	1	ВО, г. Чере	1	Растительн	4	Несколько	5	5	Нет	2	Нет ответа	0	Нет ответа
A175	0,3592	ж	2	18	162	60	22,86237	2	ВО, г. Вол	1	ВО, г. Чере	3	Рыба и ры	2	Практичес	1	12	Да	1	Нет ответа	0	Нет ответа
A172	0,0349	ж	2	18	163	63	23,71184	2	ВО, г. Чере	1	ВО, г. Чере	1	Мясные и	8	Несколько	5	5	Нет	2	Нет ответа	0	Нет ответа
A174	0,1316	ж	2	18	163	65	24,4646	2	ВО, г. Чере	1	ВО, г. Чере	1	Мясные и	28	Несколько	5	5	Да	1	Щука, леш	11	Нет ответа
A195	0,1474	ж	2	18	175	68	22,20408	2	ВО, г. Вол	1	ВО, г. Чере	3	Мясные и	1	Два-три ра	4	4	Да	1	Нет ответа	0	Нет ответа
A197	0,1666	ж	2	18	172	57	19,26717	2	ВО, Велико	2	ВО, г. Чере	3	Растительн	4	Два-три ра	2	12	Нет	2	нет ответа	0	Нет ответа
A190	0,0416	м	1	18	175	75	24,4898	2	ВО, г. Чере	1	ВО, г. Чере	1	Мясные и	1	Два-три ра	4	4	Нет	2	Нет ответа	0	Нет ответа
A191	0,1901	м	1	18	178	72	22,7244	2	ВО, г. Чере	1	ВО, г. Чере	1	Мясные и	1	Два-три ра	4	4	Нет	2	Нет ответа	0	Нет ответа
A192	0,0548	м	1	18	185	69	20,1607	2	ВО, г. Чере	1	ВО, г. Чере	1	Мясные и	20	Два-три ра	4	4	Да	1	Нет ответа	0	оз. Белое
A194	0,229	м	1	18	180	65	20,06173	2	ВО, Бабуш	2	ВО, г. Чере	3	Мясные и	8	Несколько	5	5	Да	1	Нет ответа	0	Нет ответа
A196	0,093	м	1	19	187	70	20,01773	2	ВО, г. Чере	1	ВО, г. Чере	1	Мясные и	12	Один раз в	3	3	Нет	2	Нет ответа	0	Нет ответа
A146	0,1393	ж	2	20	167	55	19,72104	2	ВО, Устюж	2	ВО, г. Чере	2	Мясные и	7	два-три ра	2	12	Да	1	Нет ответа	0	Нет ответа
A145	0,0103	м	1	21	170	80	27,68166	3	ВО, г. Чере	1	ВО, г. Чере	1	Мясные и	16	Два-три ра	4	4	Да	1	Нет ответа	0	Нет ответа
A147	0,1113	ж	2	19	162	52	19,81405	2	ВО, г. Чере	1	ВО, г. Чере	1	Мясные и	28	Несколько	5	5	Да	1	Щука, оку	16	Нет ответа
A148	0,8406	ж	2	20	175	62	20,2449	2	ВО, г. Чере	1	ВО, г. Чере	1	Рыба и ры	2	Один раз в	3	3	Да	1	Нет ответа	0	Нет ответа
A272	0,2214	м	1	18	189	80	22,39579	2	ВО, г. Чере	1	ВО, г. Чере	1	Мясные и	12	Два-три ра	4	4	Нет	2	Нет ответа	0	Нет ответа
A280	0,22	м	1	19	152	59	25,5367	3	ВО, г. Чере	1	ВО, г. Чере	1	Мясные и	1	Два-три ра	4	4	Да	1	Нет ответа	0	Нет ответа
A278	0,4589	м	1	18	185	79	23,08254	2	ВО, Белозе	2	ВО, г. Чере	2	Мясные и	39	Два-три ра	2	12	Да	1	Щука, оку	8	оз. Белое
A276	0,2273	м	1	18	174	76	25,10239	3	ВО, Бабуш	2	ВО, г. Чере	3	Мясные и	8	Два-три ра	4	4	Да	1	Нет ответа	0	Нет ответа
A275	0,1525	м	1	17	167	55	19,72104	2	ВО, Бабаев	2	ВО, г. Чере	2	Мясные и	1	Два-три ра	4	4	Да	1	Щука, оку	3	р. Суда
A269	0,1023	ж	2	18	159	52	20,56881	2	ВО, Кич-Го	2	ВО, г. Чере	3	Растительн	4	Два-три ра	4	4	Нет	2	Нет ответа	0	Нет ответа
A273	0,1039	м	1	19	178	75	23,67125	2	ВО, г. Чере	1	ВО, г. Чере	1	Мясные и	1	Несколько	5	5	Да	1	Щука, оку	3	р. Ковжа
A279	0,365	м	1	18	183	70	20,90239	2	ВО, Белозе	2	ВО, г. Чере	2	Мясные и	1	Два-три ра	2	12	Да	1	Щка, окнь	3	оз. Белое
A271	0,1212	ж	2	18	169	60	21,00767	2	ВО, г. Чере	1	ВО, г. Чере	1	Хлебобуло	3	Несколько	5	5	Нет	2	Нет ответа	0	Нет ответа
A281	0,0882	м	1	18	172	69	23,32342	2	ВО, г. Чере	1	ВО, г. Чере	1	Мясные и	7	Несколько	5	5	Да	1	Окунь, щу	3	Нет ответа
A274	0,0915	м	1	19	171	57	19,49318	2	ВО, г. Чере	1	ВО, г. Чере	1	Растительн	4	Несколько	5	5	Нет	2	Нет ответа	0	Нет ответа
A270	0,0801	ж	2	18	173	68	22,72044	2	ВО, Волог	2	ВО, г. Чере	3	Хлебобуло	3	Не употре	6	6	Нет	2	Нет ответа	0	Нет ответа
A277	0,0584	м	1	18	180	83	25,61728	3	ВО, Бабуш	2	ВО, г. Чере	3	Мясные и	1	Два-три ра	4	4	Да	1	Щука, оку	3	Нет ответа
A247	0,1012	ж	2	18	152	45	19,47715	2	ВО, г. Чере	1	ВО, г. Чере	1	Молочная	22	Два-три ра	4	4	Нет	2	Нет ответа	0	Нет ответа
A238	0,0779	ж	2	17	164	58	21,56454	2	ВО, г. Чере	1	ВО, г. Чере	1	Мясные и	7	Несколько	5	5	Нет	2	Нет ответа	0	Нет ответа
A243	0,1094	ж	2	18	169	58	20,30741	2	ВО, г. Чере	1	ВО, г. Чере	1	Мясные и	1	Два-три ра	4	4	Да	1	Нет ответа	0	Нет ответа
A237	0,2039	м	1	18	175	65	21,22449	2	ВО, г. Чере	1	ВО, г. Чере	1	Мясные и	8	Два-три ра	4	4	Да	1	Щука, оку	8	Рыбинское
A242	0,4596	ж	2	18	172	69	23,32342	2	ВО, г. Чере	1	ВО, г. Чере	1	Мясные и	20	Один раз в	3	3	Нет	2	Нет ответа	0	Нет ответа
A253	0,0288	м	1	17	Нет ответа	65			ВО, Чагод	2	ВО, г. Чере	2	Мясные и	12	Не употре	6	6	Нет	2	Нет ответа	0	Нет ответа
A248	0,9455	м	1	17	171	72	24,62296	2	ВО, г. Чере	1	ВО, г. Чере	1	Мясные и	8	Два-три ра	4	4	Да	1	Щука, окн	3	р. Юг
A240	0,0391	ж	2	18	167	Нет ответа			ВО, г. Чере	1	ВО, г. Чере	1	Мясные и	8	Не употре	6	6	Нет	2	Нет ответа	0	Нет ответа
A252	0,1518	м	1	17	174	135	44,58977	4	ВО, Шексн	2	ВО, Череп	2	Хлебобуло	6	Два-три ра	4	4	Нет	2	Нет ответа	0	Нет ответа
A239	0,0647	ж	2	18	170	48	16,609	1	ВО, г. Чере	1	ВО, г. Чере	1	Мясные и	7	Несколько	5	5	Нет	2	Нет ответа	0	Нет ответа
A251	0,1479	м	1	18	173	72	24,05693	2	ВО, г. Чере	1	ВО, г. Чере	1	Мясные и	8	два-три ра	4	4	Да	1	Щука	1	р. Суда
A250	0,1018	м	1	18	177	Нет ответа			ВО, г. Чере	1	ВО, г. Чере	1	Мясные и	1	Один раз в	3	3	Да	1	Нет ответа	0	Нет ответа
A235	0,1796	м	1	18	178	66	20,8307	2	ВО, г. Чере	1	ВО, г. Чере	1	Мясные и	8	Два-три ра	4	4	Нет	2	Нет ответа	0	Нет ответа
A246	0,1463	м	1	19	187	79	22,59144	2	ВО, г. Чере	1	ВО, г. Чере	1	Растительн	4	два-три ра	2	12	Да	1	Нет ответа	0	Нет ответа
A234	0,0232	м	1	18	177	75	23,93948	2	ВО, г. Чере	1	ВО, г. Чере	1	Мясные и	1	Несколько	5	5	Нет	2	Нет ответа	0	Нет ответа

A241	0,0679	ж	2	17	171	63	21,54509	2	ВО, г. Чере	1	ВО, г. Чере	1	Хлебобуло	3	Два-три ра	4	4	Нет	2	Нет ответа	0	Нет ответа
A245	1,686	м	1	18	181	67	20,45115	2	ВО, г. Чере	1	ВО, г. Чере	1	Мясные и	20	Два-три ра	4	4	Да	1	Щука, окун	3	Нет ответа
A249	0,1228	м	1	18	179	56	17,47761	1	ВО, г. Чере	1	ВО, г. Чере	1	Мясные и	1	Два-три ра	4	4	Нет	2	Нет ответа	0	Нет ответа
A244	0,2412	м	1	18	180	65	20,06173	2	ВО, г. Чере	1	ВО, г. Чере	1	Мясные и	1	два-три ра	2	12	Да	1	Окунь, щу	3	Нет ответа
A236	0,1827	ж	2	18	165	50	18,36547	1	ВО, г. Чере	1	ВО, г. Чере	1	Растительн	4	Два-три ра	4	4	Да	1	Щука, окун	8	Рыбинское
A294	0,0718	ж	2	18	160	56	21,875	2	ВО, г. Чере	1	ВО, г. Чере	1	Растительн	4	Два-три ра	4	4	Нет	2	Нет ответа	0	Нет ответа
A299	0,456	ж	2	18	150	51	22,66667	2	ВО, Бабаев	2	ВО, г. Чере	2	Мясные и	20	Два-три ра	4	4	Да	1	Щука, окун	3	оз. Нижнее
A300	0,14	ж	2	18	168				ВО, г. Чере	1	ВО, г. Чере	1	Мясные и	1	Несколько	5	5	Нет	2	Нет ответа	0	Нет ответа
A296	0,2143	ж	2	18	170	68	23,52941	2	ВО, г. Волс	1	ВО, г. Чере	3	Мясные и	7	Два-три ра	4	4	Нет	2	Нет ответа	0	Нет ответа
A288	0,1295	ж	2	18	162	61	23,24341	2	ВО, Никол	2	ВО, г. Чере	3	Мясные и	1	Два-три ра	4	4	Да	1	Нет ответа	0	Нет ответа
A287	0,3964	ж	2	18	173	65	21,71807	2	ВО, г. Чере	1	ВО, г. Чере	1	Растительн	4	Один раз в	3	3	Нет	2	Нет ответа	0	Нет ответа
A286	0,0628	м	1	17	185	61	17,82323	1	ВО, Чагодо	2	ВО, г. Чере	2	Мясные и	19	Не употреб	6	6	Нет	2	Нет ответа	0	Нет ответа
A292	0,1558	ж	2	18	159	47	18,59104	2	ВО, г. Чере	1	ВО, г. Чере	1	Мясные и	8	Два-три ра	2	12	Да	1	Нет ответа	0	Рыбинское
A297	0,0886	ж	2	18	163	63	23,71184	2	ВО, г. Волс	2	ВО, Черепо	2	Мясные и	20	Два-три ра	4	4	Да	1	Нет ответа	0	Нет ответа
A285	0,0347	м	1	18	184	81	23,92486	2	ВО, г. Чере	1	ВО, г. Чере	1	Мясные и	1	Несколько	5	5	Нет	2	Нет ответа	0	Нет ответа
A284	0,1291	м	1	17	179	70	21,84701	2	ВО, Черепо	2	ВО, г. Чере	2	Мясные и	20	Два-три ра	2	12	Нет	2	Нет ответа	0	Нет ответа
A290	0,0556	ж	2	18	160	49	19,14063	2	ВО, г. Чере	1	ВО, г. Чере	1	Мясные и	8	Несколько	5	5	Да	1	Нет ответа	0	р. Шексна
A289	0,127	м	1	18	200	110	27,5	3	ВО, г. Чере	1	ВО, г. Чере	1	Рыба и ры	26	Один раз в	3	3	Нет	2	Нет ответа	0	Нет ответа
A298	0,1519	ж	2	18	163	53	19,94806	2	ВО, г. Чере	1	ВО, г. Чере	1	Мясные и	8	Несколько	5	5	Да	1	Нет ответа	0	р. Шексна
A283	0,1076	ж	2	17	162	55	20,95717	2	ВО, Тотемс	2	ВО, г. Чере	3	Мясные и	8	Несколько	5	5	Да	1	Нет ответа	0	р. Сухона
A293	0,1378	ж	2	19	170	54	18,68512	2	ВО, г. Чере	1	ВО, г. Чере	1	Растительн	4	Один раз в	3	3	Да	1	Лещ	14	р. Шексна
A282	0,103	ж	2	19	163	51	19,1953	2	ВО, г. Чере	1	ВО, г. Чере	1	Мясные и	1	Два-три ра	4	4	Нет	2	Нет ответа	0	Нет ответа
A295	0,3925	ж	2	18	175	68	22,20408	2	Тверская с	1	ВО, г. Чере	1	Рыба и ры	26	Два-три ра	2	12	Да	1	Лещ, судак	15	Нет ответа
A262	0,3251	м	1	18	185	64	18,69978	2	ВО, Бабаев	2	ВО, г. Чере	2	Растительн	13	два-три ра	4	4	Да	1	Нет ответа	0	Нет ответа
A259	0,0101	м	1	18	174	77	25,43269	3	ВО, г. Чере	1	ВО, г. Чере	1	Мясные и	29	Несколько	5	5	Нет	2	Нет ответа	0	Нет ответа
A258	0,1049	м	1	18	174	57	18,82679	2	ВО, г. Чере	1	ВО, г. Чере	1	Мясные и	19	Два-три ра	4	4	Нет	2	Нет ответа	0	Нет ответа
A255	0,1635	м	1	17	187	73	20,87563	2	ВО, г. Чере	1	ВО, г. Чере	1	Мясные и	19	Два-три ра	4	4	Да	1	Щука	1	р. Маза
A268	0,0284	м	1	18	173	65	21,71807	2	ВО, Нюксе	2	ВО, г. Чере	3	Мясные и	20	Несколько	5	5	Да	1	Нет ответа	0	р. Сухона
A254	0,4362	м	1	18	181	84	25,64024	3	ВО, г. Чере	1	ВО, г. Чере	1	Мясные и	20	Два-три ра	4	4	Да	1	Щука, суда	2	р. Шексна
A266	0,0504	ж	2	18	167	62	22,23099	2	ВО, г. Чере	1	ВО, г. Чере	1	Мясные и	38	Не употреб	6	6	Нет	2	Нет ответа	0	Нет ответа
A265	0,0806	ж	2	18	156	48	19,72387	2	ВО, г. Чере	1	ВО, г. Чере	1	Мясные и	8	Не употреб	6	6	Нет	2	Нет ответа	0	Нет ответа
A257	0,2472	ж	2	17	176	70	22,59814	2	ВО, г. Чере	1	ВО, г. Чере	1	Мясные и	7	Один раз в	3	3	Нет	2	Нет ответа	0	Нет ответа
A264	0,1212	м	1	18	182	75	22,64219	2	ВО, Тотемс	2	ВО, г. Чере	3	Мясные и	19	Не употреб	6	6	Нет	1	Нет ответа	0	Нет ответа
A267	0,1814	м	1	18	177	60	19,15158	2	ВО, г. Чере	1	ВО, г. Чере	1	Растительн	4	Не употреб	6	6	Нет	2	Нет ответа	0	Нет ответа
A263	0,0401	м	1	18	174	59	19,48738	2	ВО, Чагодо	2	ВО, г. Чере	2	Мясные и	10	Не употреб	6	6	Нет	2	Нет ответа	0	Нет ответа
A261	0,0906	м	1	18	180	115	35,49383	4	ВО, г. Чере	1	ВО, г. Чере	1	Мясные и	7	Два-три ра	4	4	Нет	2	Нет ответа	0	Нет ответа
A260	0,0979	м	1	18	172	60	20,28123	2	ВО, г. Чере	1	ВО, г. Чере	1	Мясные и	28	Несколько	5	5	Нет	2	Нет ответа	0	Нет ответа
A256	0,3607	м	1	18	173	65	21,71807	2	ВО, Харово	2	ВО, г. Чере	3	Мясные и	8	Два-три ра	4	4	Да	1	Нет ответа	0	р. Кубенка
A216	0,1252	м	1	18	183	86	25,68007	3	ВО, г. Чере	1	ВО, г. Чере	1	Мясные и	7	Один раз в	3	3	Да	1	Нет ответа	0	Нет ответа
A213	0,6762	ж	2	20	168	70	24,80159	2	ВО, г. Чере	1	ВО, г. Чере	1	Мясные и	7	Несколько	5	5	Да	1	Окунь, суд	18	Нет ответа
A214	0,143	ж	2	19	173	64	21,38394	2	ВО, Велико	2	ВО, г. Чере	3	Кондитерс	5	Несколько	5	5	Нет	2	Нет ответа	0	Нет ответа
A211	0,2042	м	1	18	177	Нет ответа			ВО, Кич-Го	2	ВО, г. Чере	3	Мясные и	19	Один раз в	3	3	Нет	2	Нет ответа	0	Нет ответа
A208	0,0777	ж	2	18	158	49	19,62826	2	ВО, г. Волс	1	ВО, г. Чере	3	Хлебобуло	34	Несколько	5	5	Нет	2	Нет ответа	0	Нет ответа
A212	0,3518	ж	2	18	160	54	21,09375	2	ВО, Велико	2	ВО, г. Чере	3	Мясные и	16	Два-три ра	4	4	Нет	2	Нет ответа	0	Нет ответа
A215	0,0301	м	1	18	171	75	25,64892	3	ВО, г. Чере	1	ВО, г. Чере	1	Мясные и	20	Один раз в	3	3	Нет	2	Нет ответа	0	Нет ответа
A210	0,222	м	1	18	181	90	27,47169	3	ВО, г. Чере	1	ВО, г. Чере	1	Мясные и	20	Два-три ра	4	4	Нет	2	Нет ответа	0	Нет ответа
A228	0,1696	м	1	17	175	65	21,22449	2	ВО, Верхов	2	ВО, г. Чере	3	Мясные и	1	Несколько	5	5	Да	1	Нет ответа	0	Нет ответа
A220	0,1214	м	1	19	164	58	21,56454	2	ВО, г. Чере	1	ВО, г. Чере	1	Мясные и	20	Два-три ра	4	4	Нет	2	Нет ответа	0	Нет ответа
A217	0,4671	м	1	18	171	63	21,54509	2	ВО, Никол	2	ВО, г. Чере	3	Мясные и	25	Один раз в	3	3	Да	1	Нет ответа	0	Нет ответа
A226	0,1689	ж	2	17	168	59	20,9042	2	ВО, Бабаев	2	ВО, г. Чере	2	Хлебобуло	37	Несколько	5	5	Да	1	Окунь, щу	3	Нет ответа
A229	0,0175	м	1	18	178	59	18,62139	2	ВО, г. Чере	1	ВО, г. Чере	1	Мясные и	12	Два-три ра	4	4	Да	1	Окунь, щу	8	Рыбинское
A230	0,1334	м	1	18	183	78	23,29123	2	ВО, г. Чере	1	ВО, г. Чере	1	Молочная	22	Два-три ра	4	4	Да	1	Щука, окун	3	Нет ответа
A219	0,1114	м	1	18	174	83	27,41445	3	ВО, г. Чере	1	ВО, г. Чере	1	Растительн	4	Два-три ра	4	4	Да	1	Щука, окун	16	Анд-озеро
A218	0,1426	м	1	19	183	70	20,90239	2	ВО, г. Чере	1	ВО, г. Чере	1	Мясные и	1	Несколько	5	5	Нет	2	Нет ответа	0	Нет ответа

А221	0,1903	м	1	18	180	73	22,53086	2	ВО, Харов	2	ВО, г. Чере	3	Мясные и	36	Один раз в	3	3	Да	1	Щука, окун	3	р. Шексна
А231	0,122	м	1	18	Нет ответа	96			ВО, г. Чере	1	ВО, г. Чере	1	Мясне и к	1	два-три ра	4	4	Да	1	Нет ответа	0	Нет ответа
А222	0,1907	ж	2	18	161	44	16,97465	1	ВО, г. Чере	1	ВО, г. Чере	1	Мясные и	20	Несколько	5	5	Да	1	Нет ответа	0	р. Шексна
А225	0,3394	м	1	18	182	79	23,84978	2	Архангель	1	ВО, г. Чере	1	Мясные и	7	Один раз в	3	3	Нет	2	Нет ответа	0	Нет ответа
А224	0,031	м	1	18	169	58	20,30741	2	ВО, г. Чере	1	ВО, г. Чере	1	Мясные и	28	Один раз в	3	3	Да	1	Щука, суда	2	Нет ответа
А227	0,15	м	1	18	176	60	19,36983	2	ВО, Череп	2	ВО, г. Чере	2	Мясные и	20	Два-три ра	4	4	Да	1	Щука, окун	3	р. Суда, р.
А223	0,0372	м	1	18	175	70	22,85714	2	ВО, г. Чере	1	ВО, г. Чере	1	Раститель	4	Один раз в	3	3	Да	1	Нет ответа	0	Нет ответа
А233	0,259	м	1	19	164	62	23,05175	2	ВО, Никол	2	ВО, г. Чере	3	Мясные и	28	Два-три ра	4	4	Да	1	Щука, окун	3	р. Юг
А232	0,104	м	1	18	180	67	20,67901	2	ВО, г. Чере	1	ВО, г. Чере	1	Мясные и	19	Два-три ра	4	4	Да	1	Окунь, щу	19	Нет ответа
А332	0,1748	ж	2	18	172	90	30,42185	4	ВО, Чагод	2	ВО, г. Чере	2	Мясные и	20	Не употре	6	6	Нет	2	Нет ответа	0	Нет ответа
А335	0,2	ж	2	18	165	48	17,63085	1	ВО, Бабае	2	ВО, г. Чере	2	Мясные и	19	Два-три ра	4	4	Да	1	Нет ответа	0	Нет ответа
А337	0,1878	ж	2	18	185	47	13,73265	1	ВО, Бабае	2	ВО, г. Чере	2	Мясные и	8	Не употре	6	6	Нет	2	Нет ответа	0	Нет ответа
А327	0,3477	ж	2	18	153	45	19,22338	2	ВО, Велик	2	ВО, г. Чере	3	Крупы; мо	11	Несколько	5	5	Нет	2	Нет ответа	0	Нет ответа
А329	0,5021	ж	2	18	161	61	23,53304	2	ВО, Кирил	2	ВО, г. Чере	2	Мясные и	20	Один раз в	3	3	Да	1	Щука, окун	3	Нет ответа
А339	0,1071	ж	2	18	174	56	18,4965	2	ВО, г. Чере	1	ВО, г. Чере	1	Раститель	4	Два-три ра	4	4	Нет	2	Нет ответа	0	Нет ответа
А333	0,0695	ж	2	17	163	65	24,4646	2	ВО, Тотем	2	ВО, г. Чере	3	Мясные и	8	Два-три ра	4	4	Нет	2	Нет ответа	0	Нет ответа
А334	0,0248	ж	2	18	164	54	20,07733	2	ВО, г. Чере	1	ВО, г. Чере	1	Мясные и	1	Несколько	5	5	Нет	2	Нет ответа	0	Нет ответа
А338	0,1351	ж	2	18	158	50	20,02884	2	ВО, Верхо	2	ВО, г. Чере	3	Мясные и	12	Несколько	5	5	Нет	2	Нет ответа	0	Нет ответа
А330	0,1188	ж	2	21	160	48	18,75	2	ВО, г. Чере	1	ВО, г. Чере	1	Мясные и	20	Два-три ра	4	4	Нет	2	Нет ответа	0	Нет ответа
А328	0,1173	ж	2	18	166	61	22,13674	2	ВО, г. Вол	1	ВО, г. Чере	3	Мясные и	1	Два-три ра	4	4	Нет	2	Нет ответа	0	Нет ответа
А340	0,0898	ж	2	18	157	56	22,71897	2	ВО, г. Чере	1	ВО, г. Чере	1	Раститель	4	Не употре	6	6	Нет	2	Нет ответа	0	Нет ответа
А326	0,0783	ж	2	17	167	52	18,64534	2	ВО, Тотем	2	ВО, г. Чере	3	Мясные и	8	Два-три ра	4	4	Нет	2	Нет ответа	0	Нет ответа
А358	0,1394	ж	2	18	164	60	22,30815	2	ВО, Сямже	2	ВО, г. Чере	3	Мясные и	12	Два-три ра	4	4	Да	1	Щука, окун	3	Нет ответа
А346	0,1721	ж	2	18	155	51	21,22789	2	ВО, г. Чере	1	ВО, г. Чере	1	Мясные и	1	Два-три ра	2	12	Да	1	Нет ответа	0	Рыбинское
А353	0,0469	ж	2	18	158	49	19,62826	2	ВО, Никол	2	ВО, г. Чере	3	Мясные и	19	Не употре	6	6	Нет	2	Нет ответа	0	Нет ответа
А359	0,1343	ж	2	17	173	58	19,3792	2	ВО, Волог	2	ВО, г. Чере	3	Мясные и	20	Один раз в	3	3	Да	1	Лещ, суда	22	Нет ответа
А347	0,2347	ж	2	18	160	45	17,57813	1	ВО, Сокол	2	ВО, г. Чере	3	Мясные и	7	Два-три ра	4	4	Нет	2	Нет ответа	0	Нет ответа
А341	0,1397	ж	2	18	165	52	19,10009	2	ВО, г. Чере	1	ВО, г. Чере	1	Хлебобул	6	Несколько	5	5	Нет	2	Нет ответа	0	Нет ответа
А365	0,1736	ж	2	18	160	48	18,75	2	ВО, г. Чере	1	ВО, г. Чере	1	Мясные и	8	Несколько	5	5	Нет	2	Нет ответа	0	Нет ответа
А345	0,1716	ж	2	21	165	58	21,30395	2	ВО, г. Чере	1	ВО, г. Чере	1	Мясные и	1	Один раз в	3	3	Нет	2	Нет ответа	0	Нет ответа
А342	0,121	ж	2	18	170	67	23,18339	2	ВО, Грязов	2	ВО, г. Чере	3	Мясные и	28	Два-три ра	4	4	Нет	2	Нет ответа	0	Нет ответа
А344	0,0895	ж	2	18	165	58	21,30395	2	ВО, Вожег	2	ВО, г. Чере	2	Мясные и	12	Два-три ра	4	4	Нет	2	Нет ответа	0	Нет ответа
А362	0,09	ж	2	19	161	49	18,90359	2	ВО, Никол	2	ВО, г. Чере	3	Хлебобул	6	Не употре	6	6	Нет	2	Нет ответа	0	Нет ответа
А352	0,5508	ж	2	18	165	60	22,03857	2	ВО, Чагод	2	ВО, г. Чере	2	Хлебобул	6	Два-три ра	4	4	Да	1	Нет ответа	0	Нет ответа
А351	0,2755	ж	2	18	165	64	23,50781	2	ВО, Тотем	2	ВО, г. Чере	3	Мясные и	19	Два-три ра	4	4	Да	1	Щука, леш	11	р. Сухона
А356	0,1552	ж	2	18	167	63	22,58955	2	ВО, Тотем	2	ВО, г. Чере	3	хлебобуло	3	Несколько	5	5	Да	1	Щука, кара	13	река
А363	0,2481	ж	2	18	169	56	19,60716	2	ВО, г. Чере	1	ВО, г. Чере	1	Хлебобул	3	Два-три ра	4	4	Да	1	Нет ответа	0	Нет ответа
А360	0,2223	ж	2	17	160	48	18,75	2	ВО, Сокол	2	ВО, г. Чере	3	Мясные и	1	Два-три ра	4	4	Да	1	Щука, окун	3	р. Шексна
А364	0,0083	ж	2	18	156	45	18,49112	2	ВО, Череп	2	ВО, Череп	2	Раститель	4	Не употре	6	6	Нет	2	Нет ответа	0	Нет ответа
А355	0,2042	ж	2	18	158	63	25,23634	3	ВО, Сокол	2	ВО, г. Чере	3	Хлебобул	6	Несколько	5	5	Да	1	Нет ответа	0	оз. Кубенс
А350	0,2731	ж	2	19	174	60	19,81768	2	ВО, Белоз	2	ВО, г. Чере	2	Мясные и	20	Один раз в	3	3	Да	1	Лещ	14	оз. Белое
А361	0,1231	ж	2	18	168	63	22,32143	2	ВО, Тарно	2	ВО, г. Чере	3	Мясные и	19	Несколько	5	5	Нет	2	Нет ответа	0	Нет ответа
А354	0,2371	ж	2	18	162	42	16,00366	1	ВО, Кирил	2	ВО, г. Чере	2	Хлебобул	3	Два-три ра	4	4	Да	1	щука	1	Нет ответа
А348	0,2632	ж	2	21	172	55	18,59113	2	ВО, г. Чере	1	ВО, г. Чере	1	Мясные и	16	Два-три ра	4	4	Да	1	Щука, суда	6	Нет ответа
А305	0,0104	ж	2	18	153	43	18,369	1	ВО, г. Чере	1	ВО, г. Чере	1	Мясные и	8	Не употре	6	6	Нет	2	Нет ответа	0	Нет ответа
А302	0,6926	ж	2	18	162	54	20,57613	2	ВО, г. Чере	1	ВО, г. Чере	1	Мясные и	29	Несколько	5	5	Да	1	Щука	1	Нет ответа
А304	0,4767	ж	2	18	162	60	22,86237	2	ВО, г. Чере	1	ВО, г. Чере	1	Мясные и	40	Несколько	5	5	Да	1	Нет ответа	0	Нет ответа
А306	0,2606	ж	2	18	160	40	15,625	1	ВО, г. Чере	1	ВО, г. Чере	1	Мясные и	1	Один раз в	3	3	Нет	2	Нет ответа	0	Нет ответа
А301	0,1693	ж	2	18	160	47	18,35938	1	ВО, г. Чере	1	ВО, г. Чере	1	Мясные и	29	Несколько	5	5	Да	1	Щука	1	Нет ответа
А307	0,1397	ж	2	17	170	65	22,49135	2	ВО, Чагод	2	ВО, г. Чере	2	Хлебобул	21	Два-три ра	4	4	Да	1	Нет ответа	0	Нет ответа
А303	0,2973	ж	2	18	166	63	22,86253	2	ВО, г. Чере	1	ВО, г. Чере	1	Мясные и	1	Несколько	5	5	Да	1	Нет ответа	0	Нет ответа
А308	0,1129	ж	2	18	159	44	17,40437	1	ВО, г. Чере	1	ВО, г. Чере	1	Мясные и	7	Два-три ра	2	12	Да	1	Нет ответа	0	Нет ответа
А319	0,1782	ж	2	18	158	50	20,02884	2	ВО, г. Чере	1	ВО, г. Чере	1	Мясные и	1	Один раз в	3	3	Да	1	Щука	1	Нет ответа
А317	0,1372	ж	2	21	164	62	23,05175	2	ВО, г. Чере	1	ВО, г. Чере	1	Хлебобул	6	Один раз в	3	3	Да	1	Лещ	14	р. Суда

A324	0,0927	ж	2	20	163	75	28,22839	3	ВО, Кирил	2	ВО, г. Чере	2	Мясные и	19	Два-три ра	4	4	Да	1	Нет ответа	0	р. Шексна
A311	0,2573	ж	2	18	173	65	21,71807	2	ВО, г. Чере	1	ВО, г. Чере	1	Мясные и	12	Один раз в	3	3	Да	1	Лещ	14	р. Суда
A314	0,0766	ж	2	18	170	63	21,79931	2	ВО, Никол	2	ВО, г. Чере	3	Мясные и	1	Два-три ра	4	4	Да	1	Нет ответа	0	Нет ответа
A313	1,346	ж	2	17	168	64	22,67574	2	ВО, Вытег	2	ВО, г. Чере	2	Мясные и	28	Два-три ра	4	4	Да	1	Нет ответа	0	р. Самино
A312	0,3998	ж	2	19	164	52	19,33373	2	ВО, г. Волс	1	ВО, г. Чере	3	Хлебобуло	21	Два-три ра	4	4	Да	1	Нет ответа	0	Нет ответа
A323	0,4001	ж	2	18	160	55	21,48438	2	ВО, г. Чере	1	ВО, г. Чере	1	Мясные и	8	Два-три ра	4	4	Да	1	Щука	1	Нет ответа
A321	0,4984	ж	2	18	168	50	17,71542	1	ВО, Сокол	2	ВО, г. Чере	3	Мясные и	20	Два-три ра	4	4	Нет	2	Нет ответа	0	Нет ответа
A309	0,0799	ж	2	19	160	45	17,57813	1	ВО, г. Чере	1	ВО, г. Чере	1	Растительн	13	Не употре	6	6	Нет	2	Нет ответа	0	Нет ответа
A310	0,194	ж	2	18	168	58	20,54989	2	ВО, Тотем	2	ВО, г. Чере	3	Хлебобуло	6	Несколько	5	5	Нет	2	Нет ответа	0	Нет ответа
A408	0,1107	м	1	18	178	72	22,7244	2	ВО, Кадуй	2	ВО, г. Чере	2	Мясные и	1	Несколько	5	5	Да	1	Нет ответа	0	р. Суда
A409	0,3897	ж	2	18	158	65	26,03749	3	ВО, Усть-К	2	ВО, г. Чере	2	Растительн	4	Два-три ра	4	4	Да	1	Окунь, щу	3	р. Кубенка
A416	0,1968	ж	2	18	164	59	21,93635	2	ВО, г. Чере	1	ВО, г. Чере	1	Мясные и	20	Два-три ра	4	4	Да	1	Нет ответа	0	Рыбинское
A406	0,4298	ж	2	18	156	56	23,01118	2	ВО, г. Чере	1	ВО, г. Чере	1	Мясные и	19	Два-три ра	4	4	Да	1	Щука, суда	6	Рыбинское
A414	0,0095	м	1	19	180	84	25,92593	3	ВО, г. Чере	1	ВО, г. Чере	1	Мясные и	36	Не употре	6	6	Нет	2	Нет ответа	0	Нет ответа
A403	0,5013	ж	2	17	154	59	24,87772	2	ВО, г. Чере	1	ВО, г. Чере	1	Мясные и	8	Несколько	5	5	Да	1	Лещ	14	Нет ответа
A412	0,0852	ж	2	18	173	56	18,71095	2	ВО, Никол	1	ВО, г. Чере	1	Мясные и	29	Несколько	5	5	Нет	2	Нет ответа	0	Нет ответа
A401	0,8245	ж	2	17	165	58	21,30395	2	ВО, Вытег	2	ВО, г. Чере	2	Мясные и	28	Не употре	6	6	Нет	0	Нет ответа	0	Нет ответа
A410	0,0947	ж	2	19	151	45	19,73598	2	ВО, г. Чере	1	ВО, г. Чере	1	Хлебобуло	3	Несколько	5	5	Да	1	Нет ответа	0	Нет ответа
A420	0,0141	м	1	19	180	69	21,2963	2	ВО, г. Чере	1	ВО, г. Чере	1	Мясные и	8	Не употре	6	6	Нет	2	Нет ответа	0	Нет ответа
A411	0,0541	ж	2	18	160	51	19,92188	2	ВО, г. Чере	1	ВО, г. Чере	1	Растительн	13	Несколько	5	5	Нет	2	Нет ответа	0	Нет ответа
A419	0,0269	м	1	19	175	80	26,12245	3	ВО, г. Чере	1	ВО, г. Чере	1	Мясные и	1	Не употре	6	6	Нет	0	Нет ответа	0	Нет ответа
A407	0,6267	ж	2	18	167	51	18,28678	1	ВО, г. Чере	1	ВО, г. Чере	1	Мясные и	12	Два-три ра	4	4	Да	1	Щука, окун	3	Нет ответа
A405	0,1171	м	1	17	178	70	22,09317	2	ВО, Бабуш	2	ВО, г. Чере	3	Растительн	4	Один раз в	3	3	Да	1	Нет ответа	0	Нет ответа
A417	0,0916	ж	2	18	158	49	19,62826	2	ВО, Череп	2	ВО, г. Чере	2	Мясные и	7	Несколько	5	5	Нет	2	Нет ответа	0	Нет ответа
A404	0,1727	ж	2	18	163	50	18,81892	2	ВО, г. Чере	1	ВО, г. Чере	1	Кондитерс	5	Два-три ра	4	4	Да	1	Лещ	14	Нет ответа
A400	0,1395	ж	2	19	167	60	21,51386	2	ВО, г. Чере	1	ВО, г. Чере	1	Мясные и	1	Два-три ра	4	4	Да	1	Щука, окун	3	Нет ответа
A418	0,1434	ж	2	20	178	75	23,67125	2	ВО, Кич-Го	2	ВО, г. Чере	3	Мясные и	8	Не употре	6	6	Нет	2	Нет ответа	0	Нет ответа
A421	0,1891	ж	2	18	168	51	18,06973	1	ВО, Череп	2	ВО, Череп	2	Мясные и	28	Несколько	5	5	Да	1	Щука, окун	3	Нет ответа
A415	0,1171	ж	2	18	164	56	20,82094	2	ВО, Кич-Го	2	ВО, г. Чере	3	Мясные и	12	Два-три ра	4	4	Нет	2	Нет ответа	0	Нет ответа
A402	0,0126	ж	2	20	167	68	24,38237	2	ВО, г. Чере	1	ВО, г. Чере	1	Растительн	13	Не употре	6	6	Нет	2	Нет ответа	0	Нет ответа
A379	0,7122	ж	2	18	161	53	20,44674	2	ВО, Вожег	2	ВО, г. Чере	2	Хлебобуло	3	Два-три ра	4	4	Да	1	Щука, окун	3	оз. Воже
A383	0,4118	ж	2	18	155	46	19,14672	2	ВО, Вытег	2	ВО, г. Чере	2	Рыба и ры	41	Два-три ра	4	4	Да	1	Нет ответа	0	Нет ответа
A390	0,2309	ж	2	18	164	54	20,07733	2	ВО, Вашки	2	ВО, г. Чере	2	Растительн	13	Два-три ра	4	4	Да	1	Нет ответа	0	Нет ответа
A376	0,5069	ж	2	18	165	52	19,10009	2	ВО, Чагод	2	ВО, г. Чере	2	Мясные и	1	Один раз в	3	3	Нет	2	Нет ответа	0	Нет ответа
A377	0,0642	ж	2	19	158	50	20,02884	2	ВО, г. Чере	1	ВО, г. Чере	1	Мясные и	28	Несколько	5	5	Нет	2	Нет ответа	0	Нет ответа
A382	0,101	ж	2	18	165	50	18,36547	1	ВО, г. Чере	1	ВО, г. Чере	1	Мясные и	8	Два-три ра	4	4	Нет	2	Нет ответа	0	Нет ответа
A388	0,1674	ж	2	18	160	53	20,70313	2	ВО, г. Волс	1	ВО, г. Чере	3	Мясные и	7	Два-три ра	4	4	Нет	2	Нет ответа	0	Нет ответа
A378	0,2038	ж	2	18	175	56	18,28571	1	ВО, Череп	2	ВО, Череп	2	Мясные и	16	Один раз в	3	3	Да	1	Щука, окун	23	оз. Борода
A386	0,0977	ж	2	18	163	53	19,94806	2	ВО, г. Чере	1	ВО, г. Чере	1	Мясные и	8	Два-три ра	2	12	Да	1	Нет ответа	0	Нет ответа
A384	0,6497	ж	2	17	174	56	18,4965	2	ВО, Кич-Го	2	ВО, г. Чере	3	Мясные и	20	Два-три ра	4	4	Да	1	Щука, окун	3	р. Юг
A385	0,1523	ж	2	18	158	55	22,03173	2	ВО, Шексн	2	ВО, г. Чере	2	Мясные и	7	Два-три ра	4	4	Нет	2	Нет ответа	0	Нет ответа
A387	0,1932	ж	2	18	152	45	19,47715	2	ВО, г. Чере	1	ВО, г. Чере	1	Мясные и	8	Не употре	6	6	Нет	2	Нет ответа	0	Нет ответа
A381	0,455	ж	2	20	160	52	20,3125	2	ВО, г. Чере	1	ВО, г. Чере	1	Мясные и	16	Два-три ра	2	12	Да	1	Щука	1	Рыбинское
A389	0,0922	ж	2	18	172	65	21,97134	2	ВО, г. Чере	1	ВО, г. Чере	1	Мясные и	12	Несколько	5	5	Нет	2	Нет ответа	0	Нет ответа
A392	0,2116	ж	2	18	168	51	18,06973	1	ВО, г. Чере	1	ВО, г. Чере	1	Мясные и	20	Один раз в	3	3	Да	1	Нет ответа	0	Нет ответа
A394	0,0286	ж	2	18	156	48	19,72387	2	ВО, г. Чере	1	ВО, г. Чере	1	Мясные и	8	Несколько	5	5	Нет	2	Нет ответа	0	Нет ответа
A398	0,0553	ж	2	19	169	64	22,40818	2	ВО, г. Чере	1	ВО, г. Чере	1	Мясные и	39	Два-три ра	2	12	Нет	2	Нет ответа	0	Нет ответа
A399	0,2767	ж	2	19	165	нет ответа			ВО, г. Чере	1	ВО, г. Чере	1	Растительн	4	Один раз в	3	3	Да	1	Нет ответа	0	Нет ответа
A393	0,1581	ж	2	18	167	53	19,00391	2	ВО, г. Чере	1	ВО, г. Чере	1	Мясные и	20	Один раз в	3	3	Да	1	Щука, суда	6	Рыбинское
A396	0,2324	ж	2	18	171	54	18,46722	2	ВО, Белоз	2	ВО, г. Чере	2	Хлебобуло	3	Один раз в	3	3	Да	1	Щука, окун	23	Нет ответа
A397	0,0445	м	1	17	179	65	20,28651	2	ВО, г. Чере	1	ВО, г. Чере	1	Мясные и	1	Несколько	5	5	Нет	2	Нет ответа	0	Нет ответа
A395	0,0853	ж	2	19	162	56	21,33821	2	ВО, г. Чере	1	ВО, г. Чере	1	Мясные и	7	Два-три ра	4	4	Да	1	Щука	2	Нет ответа
A368	0,0991	ж	2	18	156	52	21,36752	2	Республик	1	ВО, г. Чере	1	Мясные и	7	Один раз в	3	3	Да	1	Нет ответа	0	Нет ответа
A372	0,6148	м	1	18	178	66	20,8307	2	ВО, г. Чере	1	ВО, г. Чере	1	Мясные и	20	Два-три ра	4	4	Да	1	Щука, окун	3	р. Суда

A371	0,1341	м	1	18	173	56	18,71095	2	ВО, Черепо	2	ВО, Черепо	2	Мясные и	1	Два-три ра	4	4	Да	1	Нет ответа	0	Нет ответа
A366	0,1078	ж	2	18	161	51	19,67517	2	ВО, г. Чере	1	ВО, г. Чере	1	Мясные и	12	Два-три ра	4	4	Нет	2	Нет ответа	0	Нет ответа
A367	0,1161	ж	2	18	155	нет ответа			ВО, г. Чере	1	ВО, г. Чере	1	Раститель	4	Два-три ра	4	4	Да	1	Нет ответа	0	река
A369	0,1614	ж	2	18	175	56	18,28571	1	ВО, г. Чере	1	ВО, г. Чере	1	Мясные и	20	Два-три ра	4	4	Да	1	Окунь	7	р. Андога
A374	0,3293	м	1	18	176,5	85	27,28535	3	ВО, г. Чере	1	ВО, г. Чере	1	Мясные и	20	Два-три ра	4	4	Да	1	Нет ответа	0	р.Шексна
A373	0,0202	м	1	21	188	80	22,63468	2	ВО, г. Чере	1	ВО, г. Чере	1	Мясные и	20	Один раз в	3	3	Нет	2	Нет ответа	0	Нет ответа
A370	0,5863	ж	2	18	178	61	19,25262	2	ВО, г. Чере	1	ВО, г. Чере	1	Мясные и	20	Два-три ра	4	4	Да	1	Нет ответа	0	Нет ответа
A375	0,0336	м	1	18	176	67	21,62965	2	ВО, г. Чере	1	ВО, г. Чере	1	Мясные и	7	Один раз в	3	3	Да	1	Щука	1	Нет ответа
A444	0,3159	ж	2	18	156	62	25,47666	3	ВО, Бабуш	2	ВО, г. Чере	3	Мясные и	12	Два-три ра	4	4	Да	1	Окунь	7	р. Сухона
A429	0,2193	ж	2	19	174	61	20,14797	2	ВО, г. Чере	1	ВО, г. Чере	1	Мясные и	19	Несколько	5	5	Нет	2	Нет ответа	0	Нет ответа
A434	0,0819	ж	2	18	165	67	24,60973	2	ВО, г. Чере	1	ВО, г. Чере	1	Мясные и	12	Несколько	5	5	Нет	2	Нет ответа	0	Нет ответа
A439	0,2335	ж	2	18	163	нет ответа			ВО, Никол	2	ВО, г. Чере	3	Хлебобуло	6	Два-три ра	2	12	Да	1	Щука, кара	13	р. Юг
A430	0,1726	ж	2	18	160	45	17,57813	1	ВО, Никол	2	ВО, г. Чере	3	Мясные и	8	Несколько	5	5	Нет	2	Нет ответа	0	Нет ответа
A425	0,15	ж	2	18	152	43	18,6115	2	ВО, г. Волс	1	ВО, г. Чере	3	Хлебобуло	3	Несколько	5	5	Да	1	Щука	1	Нет ответа
A431	0,0163	ж	2	17	170	65	22,49135	2	ВО, г. Чере	1	ВО, г. Чере	1	Мясные и	10	Не употреб	6	6	Нет	2	Нет ответа	0	Нет ответа
A432	0,0935	ж	2	18	160	55	21,48438	2	ВО, г. Чере	1	ВО, г. Чере	1	Мясные и	20	Два-три ра	4	4	Нет	2	Нет ответа	0	Нет ответа
A441	0,034	м	1	18	173	62	20,71569	2	ВО, г. Волс	1	ВО, г. Чере	3	Мясные и	1	Несколько	5	5	Нет	2	Нет ответа	0	Нет ответа
A435	0,0579	ж	2	18	154	53	22,34778	2	ВО, г. Чере	1	ВО, г. Чере	1	Мясные и	7	Несколько	5	5	Нет	2	Нет ответа	0	Нет ответа
A433	0,1404	ж	2	18	171	80	27,35885	3	ВО, Кадуйс	2	ВО, г. Чере	2	Рыба и ры	26	Один раз в	3	3	Нет	2	Нет ответа	0	Нет ответа
A423	0,1915	ж	2	18	160	54	21,09375	2	ВО, Черепо	2	ВО, Черепо	2	Мясные и	8	Несколько	5	5	Да	1	Нет ответа	0	Нет ответа
A442	0,0218	м	1	18	191	нет ответа			ВО, Черепо	2	ВО, Черепо	2	Мясные и	1	Не употреб	6	6	Нет	2	Нет ответа	0	Нет ответа
A428	0,448	ж	2	18	160	45	17,57813	1	ВО, г. Чере	1	ВО, г. Чере	1	Мясные и	16	Два-три ра	4	4	Да	1	Нет ответа	0	Нет ответа
A427	0,0639	ж	2	17	179	70	21,84701	2	ВО, Чагодо	2	ВО, г. Чере	2	Мясные и	8	Несколько	5	5	Да	1	Окунь	7	Нет ответа
A437	0,0124	м	1	17	Нет ответа	82			ВО, Чагодо	2	ВО, г. Чере	2	Мясные и	8	Не употреб	6	6	Нет	2	Нет ответа	0	Нет ответа
A443	0,1143	м	1	18	173	52	17,37445	1	ВО, Бабаев	2	ВО, г. Чере	2	Хлебобуло	6	Несколько	5	5	Нет	2	Нет ответа	0	Нет ответа
A424	0,689	ж	2	18	172	66	22,30936	2	ВО, г. Чере	1	ВО, г. Чере	1	Раститель	4	Два-три ра	4	4	Да	1	Щука	1	Нет ответа
A436	0,1336	ж	2	18	163	48	18,06617	1	ВО, Белозе	2	ВО, г. Чере	2	Хлебобуло	21	Несколько	5	5	Да	1	Нет ответа	0	оз. Белое
A438	0,0136	ж	2	18	160	45	17,57813	1	ВО, г. Чере	1	ВО, г. Чере	1	Мясные и	8	Несколько	5	5	Нет	2	Нет ответа	0	Нет ответа
A426	0,2036	ж	2	18	160	51	19,92188	2	ВО, г. Чере	1	ВО, г. Чере	1	Мясные и	16	Один раз в	3	3	Нет	0	Нет ответа	0	Нет ответа
A450	0,0586	ж	2	18	181	60	18,31446	1	ВО, г. Чере	1	ВО, г. Чере	1	Мясные и	19	Несколько	5	5	Да	1	Окунь, щу	3	оз. Ильмен
A448	0,1584	ж	2	18	181	нет ответа			ВО, г. Чере	1	ВО, г. Чере	1	Мясные и	19	Два-три ра	4	4	Да	1	Щука, суда	2	Нет ответа
A447	0,0828	ж	2	19	178	нет ответа			ВО, г. Чере	1	ВО, г. Чере	1	Раститель	13	Два-три ра	4	4	Да	1	Окунь, щу	8	Нет ответа
A449	0,2561	ж	2	18	163	59	22,20633	2	ВО, Кадуйс	2	ВО, г. Чере	2	Хлебобуло	6	Один раз в	3	3	Да	2	Нет ответа	0	Нет ответа
A446	0,0201	м	1	18	185	75	21,91381	2	ВО, Черепо	2	ВО, Черепо	2	Мясные и	8	Два-три ра	4	4	Да	1	Щука, окун	3	Рыбинское
A445	0,2208	ж	2	17	167	58	20,79673	2	ВО, г. Чере	1	ВО, г. Чере	1	Мясные и	19	Два-три ра	4	4	Да	1	Нет ответа	0	Нет ответа
